# SARDH in the 1-C metabolism sculpts the T-cell fate and serves as a potential cancer therapeutic target

**DOI:** 10.1038/s41423-025-01331-5

**Published:** 2025-08-20

**Authors:** Wen Si, Sijin Cheng, Haiyin He, Yu Zhang, Yuhui Miao, Dingcheng Yi, Mengjiao Ni, Anqiang Wang, Hongtao Fan, Yufei Bo, Chang Liu, Zhaode Bu, Linnan Zhu, Zemin Zhang

**Affiliations:** 1 Biomedical Pioneering Innovation Center (BIOPIC) and School of Life Sciences, State Key Laboratory of Metabolic Dysregulation & Prevention and Treatment of Esophageal Cancer, Peking University, Beijing, China; 2 Academy for Advanced Interdisciplinary Studies, Peking University, Beijing, China; 3 Institute for Data-Driven Tumor Immunology, Chongqing Medical University, Chongqing, China; 4 Changping Laboratory, Beijing, China; 5 Gastrointestinal Cancer Center, Key Laboratory of Carcinogenesis and Translational Research (Ministry of Education), Peking University Cancer Hospital and Institute, Beijing, China; 6 State Key Laboratory of Holistic Integrative Management of Gastrointestinal Cancers, Beijing Key Laboratory of Carcinogenesis and Translational Research, Department of Gastrointestinal Cancer Center, Peking University Cancer Hospital & Institute, Beijing, China

**Keywords:** Mitochondrial metabolism, Tumor microenvironment, CD8^+^ exhausted T cells, 1-C metabolism, SARDH, Sarcosine, Immunosuppression, Cancer immunotherapy, Cancer metabolism

## Abstract

T-cell metabolism plays a pivotal role in defining T-cell functional states. Through analysis of a comprehensive pancancer single-cell transcriptional atlas, we identified SARDH, an enzyme involved in one-carbon (1-C) metabolism, as a potential T-cell metabolic checkpoint. SARDH significantly impacts T-cell fate and function, leading to impaired tumor control efficacy. Knocking down SARDH resulted in sarcosine accumulation and reduced consumption of S-adenosylmethionine (SAM), a critical methyl donor for epigenetic modulation, likely due to the shift in glycine-to-sarcosine homeostasis. Deletion of SARDH increased H3K79me2 modification at NF-κB-activating genes, thereby augmenting NF-κB signaling and T-cell function. Additionally, we observed transcriptional dysregulation of 1-C metabolism within tumors across various cancer types, which was often accompanied by increased sarcosine levels. Sarcosine was found to induce *SARDH* upregulation, suggesting a feedback mechanism for metabolic homeostasis in T cells within tumors. These findings underscore the potential effects and mechanism of targeting 1-C metabolism, particularly SARDH, as an avenue for cancer therapy.

## Introduction

Immunotherapy has dramatically transformed cancer treatment [1–3], and T cells are one of the focal points in the development of immune therapeutics [2, 4, 5]. However, a major challenge that compromises antitumor efficacy is T-cell dysfunction, which is induced by the tumor microenvironment (TME) [6, 7]. Although current strategies targeting immune checkpoints such as PD-1 have made strides, they still fail to sufficiently ameliorate T-cell exhaustion [8, 9]. This limitation is further exacerbated by patient heterogeneity, leading to only a subset benefiting from these therapies [10–12]. Consequently, the implementation of pancancer screening for more effective and universal drug targets that can alleviate inhibitory constraints and reverse T-cell dysfunction is highly desirable in the context of cancer immunotherapy.

The importance of metabolism in T cells is increasingly recognized [13–16]. In addition to TCR signals, costimulatory and coinhibitory signals, and cytokine signals, the activation of metabolic pathways is considered essential as “signal 4” for the T-cell response [14, 17, 18]. Mitochondria, central metabolic organelles, are involved in various cellular processes, including energy production, biosynthesis, and signaling molecule generation [19]. To some extent, cancer can be considered a mitochondrial metabolic disease [20, 21]. Mitochondria play crucial roles in orchestrating T-cell fate and functions [16], with their metabolism being reshaped in the TME due to nutrient and oxygen scarcity, as well as metabolites secreted by other cells [13]. These alterations can lead to T-cell exhaustion, consequently inhibiting antitumor activity [13, 14, 22–24]. In turn, reprogrammed mitochondrial functions have also been regarded as features of T-cell exhaustion [24]. However, the mechanisms through which mitochondrial metabolism impacts T-cell fate and function remain unclear, underscoring the need for further comprehensive exploration of T-cell mitochondrial metabolism within the TME.

Single-cell RNA sequencing (scRNA-seq) has revolutionized our ability to dissect the intricate cellular landscape and intercellular interactions, thereby facilitating a systematic understanding of tumors [25, 26]. With its widespread application, sizable single-cell transcriptomic data for cellular components inside tumors have been generated [27–29]. We previously constructed a pancancer single-cell atlas of T cells, laying the foundation for an in-depth characterization of the mitochondrial metabolic features of tumor-infiltrating T cells at the transcriptome level [10]. In this study, we conducted a comprehensive analysis of the metabolic adaptation of T cells inside tumors. We established a computational screening pipeline focused on mitochondria-related genes and identified sarcosine dehydrogenase (SARDH) as a potential key regulator of T-cell mitochondrial metabolism in multiple cancer types. SARDH, a mitochondrial flavoenzyme, plays an important role in one-carbon (1-C) metabolism [30], which appears to be reprogrammed in both tumor and infiltrating T cells. Through both in vitro and in vivo experiments, we discovered that SARDH acts as a potential metabolic checkpoint that restrains T-cell functions. We propose an H3K79me2-related mechanism wherein SARDH exerts its suppressive effect by inhibiting the nuclear factor kappa B (NF-κB) signaling pathway through regulating the homeostasis of related metabolites. Notably, our findings suggested that SARDH expression was inducible by its substrate, sarcosine, which was inferred to accumulate in tumors. Overall, our study emphasized the potential therapeutic implications of targeting SARDH in T-cell-based cancer immunotherapy.

## Results

### Pancancer analysis of tumor-infiltrating T cells revealed that the mitochondrial metabolic gene *SARDH* is related to CD8^+^ T-cell dysfunction

**Fig. 1:**
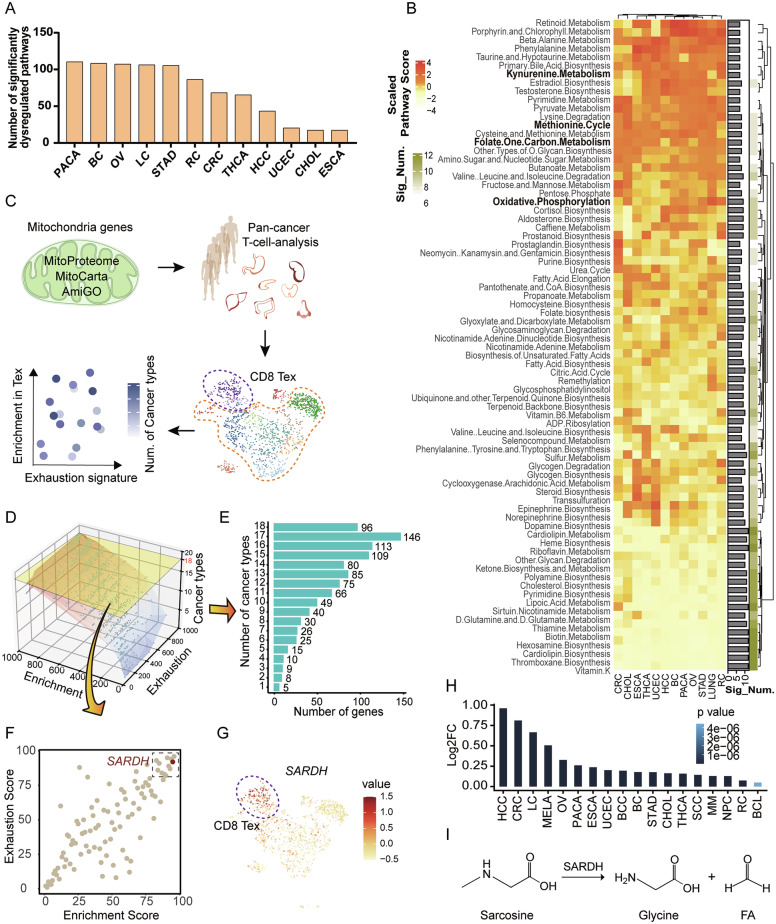
Pancancer analysis of tumor-infiltrating T cells reveals that the mitochondrial metabolic gene *SARDH* is related to CD8^+^ T-cell dysfunction. **A** Bar plot illustrating the number of significantly dysregulated pathways across each cancer type. **B** Heatmap illustrating the scaled pathway score and the number of significantly dysregulated pathways across each cancer type. **C** Scheme of the computational analysis workflow for screening candidate mitochondrial metabolic genes. **D** Three-dimensional plot showing all mitochondria-related genes with enrichment scores, exhaustion scores, and numbers of cancer types in which genes were significantly upregulated in exhausted T cells. **E** Bar plot showing the number of mitochondria-related genes significantly upregulated in exhausted T cells among different numbers of cancer types. **F** Scatterplot showing that the enrichment score and exhaustion score of mitochondria-related genes were significantly increased in all 18 collected cancer types. **G** UMAP plot showing *SARDH* expression in CD8^+^ T cells. **H** Bar plot showing log_2_ (fold change) values of *SARDH* in exhausted T cells across each cancer type. **I** Enzymatic reaction of sarcosine decomposition catalyzed by SARDH. The cancer type abbreviations used are as follows: BCC basal cell carcinoma, BC breast cancer, BCL B-cell lymphoma, CHOL cholangiocarcinoma, CRC colorectal cancer, ESCA esophageal carcinoma, HCC hepatocellular carcinoma, LC lung cancer, MELA melanoma, MM multiple myeloma, NPC nasopharyngeal cancer, OV ovarian cancer, PACA pancreatic cancer, RC renal carcinoma, SCC squamous cell carcinoma, STAD stomach adenocarcinoma, THCA thyroid carcinoma, UCEC uterine corpus endometrial carcinoma

The metabolism of tumor-infiltrating T cells is known to be notably reprogrammed, profoundly impacting T-cell functionality [14, 31]. To dissect this complex metabolic reprogramming process, we performed a pancancer transcriptional comparison of 114 key metabolic pathways contained in the public database between T cells isolated from tumors and those from corresponding normal tissues across 12 cancer types [10, 32]. This analysis revealed significant transcriptional dysregulation in many metabolic pathways, particularly in breast cancer (BC), lung cancer (LC), ovarian cancer (OV), pancreatic cancer (PACA), and stomach adenocarcinoma (STAD) (Fig. 1A). Remarkably, 77 out of the 114 surveyed metabolic pathways exhibited significant alterations in more than half of the cancer types studied (Fig. 1B; Supplementary Fig. S1A).

Notably, certain dysregulated pathways appeared to be linked to T-cell exhaustion in the TME (Fig. 1B). For example, oxidative phosphorylation in mitochondria is known to be impaired during persistent antigenic stimulation in tumors, leading to reduced proliferation and aggravated exhaustion in T cells [33]. As another example, the metabolism of kynurenine—a byproduct of tryptophan catabolism resulting from the high expression of indoleamine 2,3-dioxygenase 1 (IDO1) in cancer cells—has been shown to suppress the cytotoxic functions of CD8^+^ T cells, ultimately leading to T-cell exhaustion [34]. These observations suggest that other dysregulated metabolic pathways in tumor-infiltrating T cells might similarly affect T-cell fate and function.

To further explore the metabolic underpinnings of T-cell exhaustion, we developed a computational pipeline to analyze single-cell RNA sequencing (scRNA-seq) data, focusing on metabolic genes in exhausted CD8^+^ T cells within the TME (Fig. 1C). Given the central role of mitochondria in cell energy production and biological synthesis, which are especially vital for the fate and function of T cells in tumors, we focused our screening efforts on mitochondrial metabolic genes [16, 35]. Using data from MitoCarta [36], MitoProteome [37], and AmiGO [38], we identified a total of 1264 mitochondria-related genes (Supplementary Fig. S1B). We then conducted a detailed examination of the expression characteristics of these mitochondria-related genes in tumor-infiltrating T cells via our previously established pancancer single-cell transcriptome atlas, which included 215,444 T cells from 238 patients across 18 cancer types [10]. We systematically prioritized the mitochondria-related genes specifically enriched in exhausted CD8^+^ T cells on the basis of three key criteria: 1) high universality across different cancer types, 2) a significant fold change in expression within exhausted T cells compared with the other subtypes (referred to as the enrichment score), and 3) a strong correlation with T-cell exhaustion markers (referred to as the exhaustion score) (Fig. 1D). Despite the inherent heterogeneity between various cancer types, we identified 96 mitochondria-related genes that were significantly upregulated in exhausted CD8^+^ T cells universally (the highest universality; Fig. 1E). Among these, we focused on genes with high enrichment and exhaustion scores as potential candidates influencing T-cell dysfunction (Fig. 1F; Supplementary Fig. S1C, D).

Given the high conservation of mitochondrial processes across cell types, we sought to identify target genes for therapeutic intervention without adversely affecting other tissues. To address this, we cross-checked the Human Protein Atlas to examine the expression profiles of our candidate genes across various cell types [39], aiming for those with restricted expression patterns and significant enrichment in T cells.

Ultimately, our combined analyses pinpointed *SARDH* as the top gene for investigating its potential influence on T-cell dysfunction. SARDH is a mitochondrial flavoenzyme involved in 1-C metabolism and catalyzes the conversion of sarcosine to glycine [40]. The upstream metabolic pathways related to SARDH, such as the methionine cycle and folate one-carbon metabolism, were systemically dysregulated in tumor-infiltrating T cells, suggesting that 1-C metabolism was reprogrammed during the adaptation of T cells in the TME [30] (Fig. 1B). Notably, *SARDH* was upregulated in exhausted CD8^+^ T cells across all cancer types (Fig. 1G, H; Supplementary Fig. S1E) and displayed a robust correlation with exhaustion markers, outperforming most other mitochondria-related genes (Fig. 1F; Supplementary Fig. S1C, D). Furthermore, *SARDH* was significantly enriched within the IFNG^+^ Tfh/Th1 group among the CD4^+^ T-cell subtypes (Supplementary Fig. S1F), which exhibited high exhaustion marker expression. Additionally, *SARDH* demonstrated greater specificity for T cells, coupled with a relatively confined expression profile across normal tissues (Supplementary Fig. S1G, H). Taken together, these findings collectively suggest that SARDH potentially serves as a mitochondrial metabolic gene associated with T-cell dysfunction.

### SARDH restricts T-cell activation, proliferation, and cytotoxic function

**Fig. 2:**
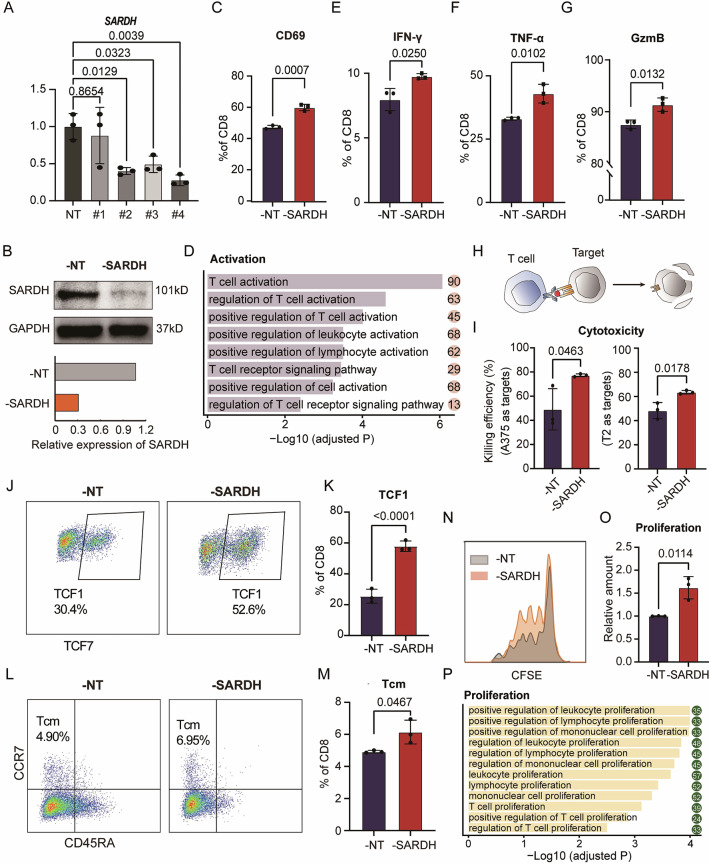
SARDH restricts T-cell activation, proliferation and cytotoxic function. **A** Bar plot illustrating the relative expression levels of *SARDH* in T cells treated with 4 siRNAs targeting *SARDH* (#1‒#4) at different sites compared with those in T cells treated with nontargeting siRNA (NT). *n* = 3. **B** Western blot analysis of SARDH expression in nontargeting control and SARDH-knockdown human T cells. Bar plot showing the densitometric analysis, which quantifies SARDH normalized to GAPDH. **C** Bar plot depicting the proportion of CD69**-**expressing cells in nontargeting control and SARDH-knockdown human CD8^+^ T cells. *n* = 3. **D** Bar plot showing activation-related GO terms enriched in SARDH-knockdown samples compared with nontargeting controls, with gene counts indicated within solid circles. **E**–**G** Bar plots depicting the proportions of nontargeting control and SARDH-knockdown human CD8^+^ T cells expressing T-cell function-related markers, including IFN-γ (**E**), TNF-α (**F**), and granzyme B (**G**). *n* = 3. **H** Schematics illustrating the in vitro T-cell cytotoxicity analysis. **I** quantified the killing efficiency and revealed the cytotoxicity of the nontargeting control and SARDH-knockdown CD8^+^ T cells to A375 or T2 target cells. *n* = 3. **J‒M** Representative FACS plots illustrating the percentages of TCF-1-expressing (**J**) and Tcm-expressing (**L**) nontargeting control and SARDH-knockdown human CD8^+^ T cells. Quantifications of the proportions of TCF-1 (**K**) and Tcm (**M**) are also presented. *n* = 3. **N** FACS histogram showing fluorescence intensity distributions in CFSE-stained nontargeting control and SARDH-knockdown human CD8^+^ T cells. **O** Relative quantification of the proliferation abilities of nontargeting control and SARDH-knockdown human CD8^+^ T cells. *n* = 3. **P** Bar plot showing proliferation-related GO terms enriched in the SARDH-knockdown samples compared with the nontargeting controls. The numbers on the right indicate gene counts matched to the corresponding terms. All the data from the FACS analysis are presented as the means with SDs and are representative of at least three independent experiments. All statistically significant differences were calculated via an unpaired *t* test

To investigate the effects of SARDH on T-cell fate and function, we used RNA interference (RNAi) to knock down SARDH expression in T cells (Fig. 2A, B; Supplementary Fig. S2A–C). Knocking down SARDH did not result in significant changes in the mitochondrial membrane potential, suggesting that mitochondrial function was preserved (Supplementary Fig. S2D). We observed that T cells with reduced SARDH expression exhibited increased activation, as indicated by increased CD69 expression (Fig. 2C; Supplementary Fig. S3A, B). Gene Ontology (GO) enrichment analysis of the transcriptomic data further emphasized the suppressive role of SARDH in activation-related pathways (Fig. 2D; Supplementary Fig. S3C). Furthermore, downregulation of SARDH in CD8^+^ T cells led to increased production of crucial cytokines, such as IFN-γ and TNF-α, as well as the effector molecule granzyme B (Fig. 2E–G; Supplementary Fig. S3D, E, H). A similar inhibitory effect of SARDH on cytokine secretion was also observed in CD4^+^ T cells (Supplementary Fig. S3D–G).

The observed effects of SARDH ultimately resulted in the inhibition of T-cell cytolytic function. To evaluate the impact of SARDH on T-cell-specific killing ability, we generated MART-1-specific T-cell receptor (TCR)-engineered T (TCR-T) cells and cocultured them with target cells (Fig. 2H; Supplementary Fig. S4A). T cells with decreased expression of SARDH exhibited increased killing efficiency against MART-1-positive (MART-1^+^) A375 cells and MART-1_27-35_-pulsed T2 cells (Fig. 2I). In contrast, overexpression of SARDH impaired the cytotoxic activity of MART-1-specific TCR-T cells, resulting in a higher survival rate of A375 target cells in coculture (Supplementary Fig. S4B–E).

Interestingly, despite the enrichment of *SARDH* in exhausted CD8^+^ T cells, it had no significant effect on the expression of molecules that are typically employed to indicate exhausted T cells at both the RNA and protein levels (Supplementary Fig. S5A, B). However, our data indicated that SARDH appeared to play a role in modulating CD8^+^ and CD4^+^ T-cell stemness, as its knockdown led to an increased proportion of TCF1^+^ cells and central memory T cells (CD45RA^−^CCR7^+^; Tcm), indicating a suppressive role of SARDH in sustaining immune responses (Fig. 2J–M; Supplementary Fig. S5C–G). Additionally, SARDH was found to restrain the proliferation of both CD8^+^ and CD4^+^ T cells. Carboxyfluorescein succinimidyl ester (CFSE) staining indicated that CD8^+^ and CD4^+^ T cells with reduced SARDH expression were more active in terms of cell division, leading to an increased cell number under similar culture conditions (Fig. 2N, O; Supplementary Fig. S5H, I). Transcriptomic GO enrichment analysis confirmed the suppressive role of SARDH in proliferation-related pathways (Fig. 2P). Taken together, these in vitro findings suggest that SARDH plays a significant inhibitory role in multiple T-cell functions.

### SARDH inhibits the spatial migration of T cells

**Fig. 3:**
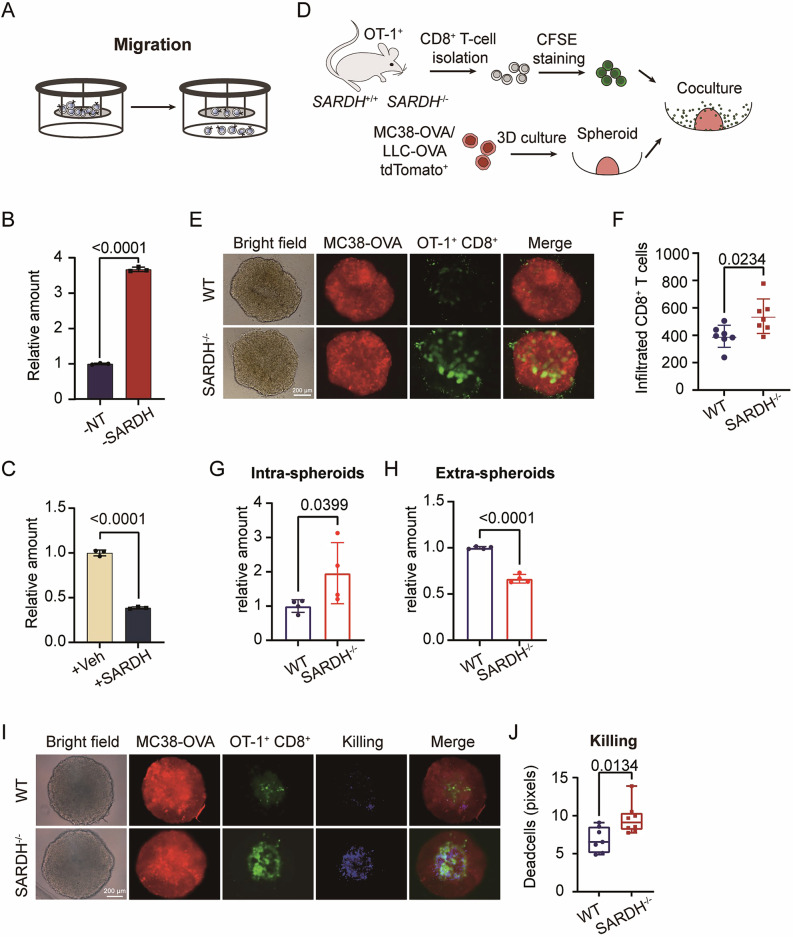
SARDH inhibits the spatial migration of T cells. **A** Schematic of the measurement of the migration ability of human T cells via the transwell assay. **B** Bar plot showing the status of human T-cell migration after 24 h in both the nontargeting control and SARDH knockdown groups. *n* = 3. **C** Status of human T-cell migration after 24 h in both the control (Vec) and SARDH-overexpressing (+SARDH) groups. *n* = 3. **D** Schematics illustrating the tumor spheroid-T-cell coculture assay. **E** Bright-field and multispectral fluorescence imaging of a representative coculture system after 48 h of coculture with tdTomato^+^ MC38-OVA spheroids (red) and staining SARDH^+/+^ or SARDH^−/−^ OT-1^+^ CD8^+^ T cells (green). **F** Scatter dot plot illustrating the infiltration of SARDH^+/+^ or SARDH^−/−^ OT-1^+^ CD8^+^ T cells into MC38-OVA spheroids. *n* = 7. **G**–**H** Bar plots showing the relative numbers of both infiltrating (**G**) and noninfiltrating (**H**) OT-1^+^ CD8^+^ T cells with or without SARDH knockout. **I** Bright-field and multispectral fluorescence images after 48 h of coculture showing tdTomato^+^ MC38-OVA spheroids (red), OT-1^+^ CD8^+^ T cells (green) and dead cells (blue) in the SARDH^+/+^ and SARDH^−/−^ groups. **J** Box-and-whisker plot illustrating the tumor-cell-killing abilities of SARDH^+/+^ or SARDH^−/−^ OT-1^+^ CD8^+^ T cells. SARDH^+/+^, *n* = 7; SARDH^−/−^, *n* = 8. All the bar plot data are presented as the means with SDs, and all the scatter dot plot data are presented as the medians with interquartile ranges. All the data shown are representative of at least three independent experiments. All significances presented were calculated via an unpaired *t* test

The migration and infiltration capabilities of T cells are essential for the effective elimination of cancer cells, enabling their direct access to tumor sites [41, 42]. We investigated the effects of SARDH on T-cell migration with a transwell assay (Fig. 3A). Silencing of *SARDH* significantly enhanced T-cell migration through the Matrigel layer, a substrate that mimics the tumor matrix (Fig. 3B; Supplementary Fig. S6A). In contrast, overexpression of SARDH reduced the ability of T cells to migrate through the matrix (Fig. 3C). GO enrichment analysis revealed that SARDH negatively impacts cell‒cell adhesion, a key factor for cell motility [42] (Supplementary Fig. S6B). These findings suggest that SARDH limits T-cell migration, potentially impairing their ability to infiltrate tumors and perform antitumor activities.

To explore the impact of SARDH on T-cell migration within the tumor microenvironment, we employed three-dimensional tumor spheroid models using ovalbumin-positive MC38 (MC38-OVA) and LLC-OVA cell lines. OT-1^+^ CD8^+^ T cells derived from both wild-type (WT) and SARDH knockout (SARDH^−/−^) OT-1^+^ mice were introduced into these tumor spheroids (Fig. 3D). Compared with WT CD8^+^ T cells, SARDH^−/−^ CD8^+^ T cells exhibited greater infiltration into the tumor spheroids in both the MC38- and LLC-derived spheroid models (Fig. 3E, F; Supplementary Fig. S6C, D). To confirm that the increased infiltration was not confounded by enhanced T-cell proliferation due to SARDH depletion, we quantified both infiltrated and noninfiltrated T cells in the MC38-spheroid model. SARDH^−/−^ T cells accounted for a greater proportion of infiltrated cells, accompanied by a relatively lower number of T cells outside of the spheroid, suggesting that the observed difference was attributed to increased T-cell infiltration (Fig. 3G, H). Moreover, SARDH depletion enhanced the effectiveness of T-cell-mediated killing by MC38-OVA within the spheroids (Fig. 3I, J).

We further generated human cell line-derived tumor spheroids from MART-1^+^ A375 cells and incubated them with MART-1-specific TCR-T cells to investigate the role of SARDH in human T-cell infiltration. Upon the knockdown of SARDH in these T cells, we observed an increase in T-cell infiltration into human cell line-derived tumor spheroids (Supplementary Fig. S6E, F). We subsequently assessed the effectiveness of T-cell infiltration in tumor organoids derived from colorectal cancer (CRC) patients. We labeled the organoids and syngeneic T cells isolated from peripheral blood with different fluorochromes and cocultured them together (Supplementary Fig. S6G, H). In a model that more closely mimics the tumor microenvironment, where the proportion of tumor-reactive T cells in the peripheral blood is lower, we still observed that knocking down SARDH enhanced T-cell infiltration, as expected (Supplementary Fig. S6I, J). In summary, our findings underscore the critical role of SARDH in regulating T-cell migration and infiltration into tumor sites, ultimately dampening its efficacy in tumor suppression.

### SARDH deficiency in T cells delays tumor progression in vivo

**Fig. 4:**
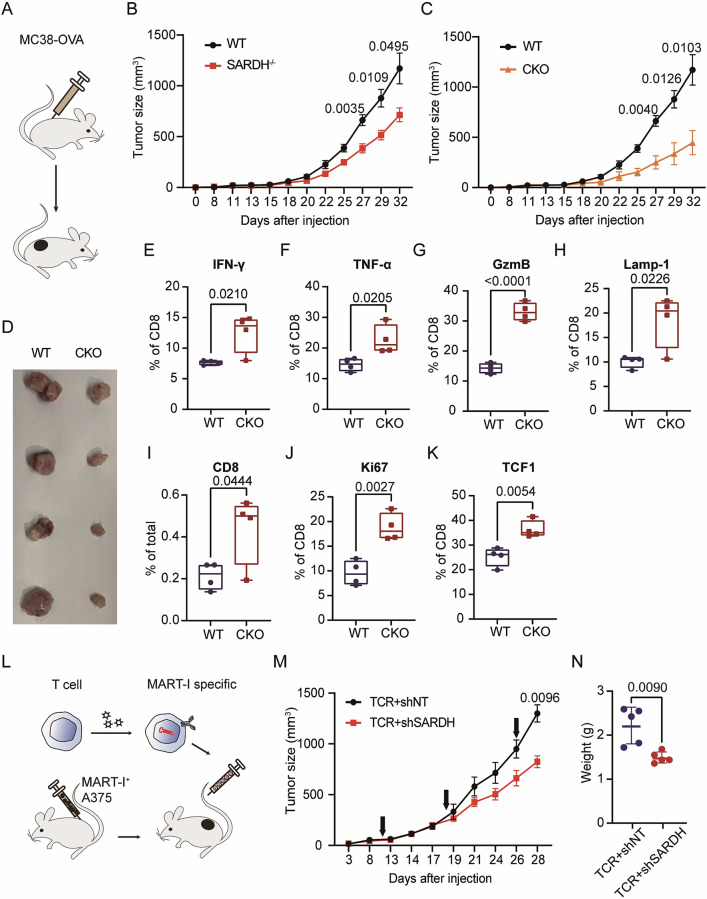
SARDH deficiency in T cells delays tumor progression in vivo. **A** Schematic illustrating the subcutaneous tumor-bearing experiments. **B** Tumor growth curve over days postinjection of MC38-OVA cells into WT and SARDH^−/−^ OT-1^+^ mice. WT: *n* = 6; SARDH^−/−^: *n* = 10. **C** Tumor growth curve over days postinjection of MC38-OVA cells into WT and CKO OT-1^+^ mice. WT: *n* = 6; CKO: *n* = 4. **D** Representative image of tumors from WT mice and CKO OT-1^+^ mice used for the analysis of tumor-infiltrating T cells. **E–H **Box‒and‒whisker plots showing the proportions of cells expressing cell function-related markers, including IFN-γ (**E**), TNF-α (**F**), granzyme B (**G**), and Lamp-1 (**H**), among tumor-infiltrating CD8^+^ T cells isolated from WT and CKO mice. *n* = 4. **I** Box-and-whisker plot showing the relative quantification of CD8^+^ T-cell percentages in WT and CKO mouse tumors. *n* = 4. **J** Proportions of Ki67-expressing CD8^+^ T cells in WT and CKO mice. *n* = 4. **K** Proportions of TCF-1-expressing CD8^+^ T cells in WT and CKO mice. *n* = 4. **L** Schematic showing that NSG mice were inoculated with MART-1^+^ A375 cells and subsequently with MART-1-specific T cells at certain time points. **M** Tumor growth curve over days postinjection with MART-1^+^ A375. The arrows indicate the time points at which TCR-T cells were injected. *n* = 5. **N** Scatter dot plot illustrating the weight of tumors after injection of TCR-T cells transfected with non-targeting shRNA (shNT) or shRNA targeting *SARDH* (shSARDH). *n* = 5. All the bar plot data are presented as the means with SDs, all the growth curve data are presented as the means with SEMs, and all the scatter dot plot data are presented as the medians with interquartile ranges. All the data shown are representative of at least three independent experiments. All significances presented were calculated via an unpaired *t* test

To further assess the impact of SARDH on T-cell function and its potential to impair tumor control, we conducted experiments using both systemic and conditional SARDH knockout OT-1^+^ mice that specifically recognize MC38-OVA. We analyzed the tumor progression rates of subcutaneous MC38-OVA in SARDH^−/−^ OT-1^+^ mice (Fig. 4A). Tumor growth was notably inhibited in the SARDH^−/−^ OT-1^+^ mice compared with the WT OT-1^+^ mice (Fig. 4B; Supplementary Fig. S7A‒C). Similarly, we observed markedly slower tumor growth in mice with T-cell-specific knockout of SARDH (Lck-CRE, SARDH^F/F^, OT-1^+^; CKO) than in WT mice (Fig. 4C; Supplementary Fig. S7A‒C). These data highlight a potential T-cell-mediated mechanism of tumor control.

To further investigate how SARDH influences this process, we harvested and analyzed subcutaneous tumors from CKO and WT mice, focusing on the characteristics of infiltrating T cells, particularly CD8^+^ T cells (Fig. 4D). We observed elevated secretion of cytokines such as TNF-α and IFN-γ, along with increased degranulation, as indicated by the expression of granzyme B and Lamp-1 in CD8^+^ T cells from tumors in CKO mice (Fig. 4E–H; Supplementary Figs. S7D, S8A–D, G–J). These alterations induced by SARDH knockout in CD8^+^ T cells partially contributed to the enhanced tumor control ability.

Additionally, the infiltration of both CD4^+^ and CD8^+^ T cells within tumors in CKO mice improved, potentially assisting in the effective function of these cells within the tumor microenvironment (Fig. 4I; Supplementary Fig. S7E–G). Moreover, infiltrating CD8^+^ T cells in CKO mice also exhibited increased proliferation, as indicated by a higher Ki67^+^ ratio (Fig. 4J; Supplementary Fig. S8E, K) and a greater proportion of TCF1^+^ cells, suggesting increased stemness in vivo (Fig. 4K; Supplementary Fig. S8F, L). However, the expression of exhaustion markers was not obviously altered with SARDH knockout (Supplementary Fig. S9A–C). Taken together, these findings suggest that SARDH may serve as a metabolic checkpoint for optimizing T-cell effector function in vivo.

To assess the clinical potential of targeting SARDH in TCR-T-cell therapy, which holds significant promise in cancer treatment [5, 43], we established a cell-derived xenograft model by inoculating MART-1^+^ A375 cells into NOD. Cg-*Prkdc*^scid^*Il2rg*^em1Smoc^ (NSG) immunodeficient mice, followed by injection of MART-1-specific TCR-T cells via the tail vein (Fig. 4L). TCR-T cells with reduced SARDH levels exhibited enhanced inhibition of A375 tumor progression (Fig. 4M, N). We observed no significant change in the expression of coinhibitory receptors, including PD-1, TIM-3, and LAG-3, in CD8^+^ T cells upon SARDH knockdown in this in vivo model, further indicating that there was no obvious alteration in the exhaustion state (Supplementary Fig. S9D, E). Collectively, these results suggest that targeting SARDH could serve as a potential target to increase the efficacy of T-cell-based cancer therapies.

### SARDH suppresses T-cell function through metabolite modulation

**Fig. 5:**
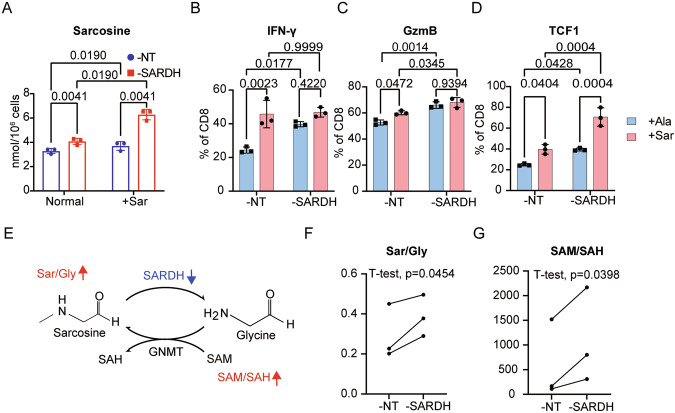
SARDH suppresses T-cell function through metabolite modulation. **A** Concentrations of sarcosine detected in nontargeting control and *SARDH*-knockdown human CD8^+^ T cells cultured in normal medium or supplemented with sarcosine (+Sar). *n* = 3. **B–D** Bar plots showing the percentages of human CD8^+^ T cells expressing IFN-γ (**B**), granzyme B (**C**) and TCF-1 (**D**) in the nontargeting control and SARDH knockdown groups cultured with supplemented sarcosine (+Sar) or the control amino acid alanine (+Ala). *n* = 3. **E** Enzymatic reactions catalyzed by SARDH and GNMT. **F**–**G** Scatterplots illustrating the ratios of sarcosine to glycine (**F**) and SAM to SAH (**G**) in nontargeting control and *SARDH*-knockdown human CD8^+^ T cells. The significance presented was calculated via a paired one-sided *t* test. *n* = 3. All the bar plot data are presented as the means with SDs. All the data shown are representative of at least three independent experiments. All significances presented in the bar plots were calculated via an unpaired *t* test

To explore how SARDH affects T-cell fate and function, we hypothesized that it regulates T-cell signaling via its enzymatic activity, potentially through modulating the concentrations of relevant metabolites. Since SARDH is known to convert sarcosine into glycine, we examined the enzymatic effects of SARDH knockdown on this process. Knocking down SARDH led to increased sarcosine levels in T cells, as observed both in standard medium and in medium supplemented with 100 nM sarcosine (Fig. 5A). These results demonstrated that SARDH regulates the sarcosine concentration. When T cells were cultured under high-sarcosine conditions to mimic SARDH knockdown, they exhibited better survival, accelerated growth and enhanced cytotoxicity, as evidenced by increased IFN-γ and granzyme B production in CD8^+^ T cells (Fig. 5B, C; Supplementary Fig. S10A–C). Moreover, the stemness of CD8^+^ T cells was better maintained, as indicated by the proportion of TCF1^+^ cells (Fig. 5D). These results indicated that elevated sarcosine levels resulting from reduced SARDH could drive improvements in T-cell fate and function.

Since SARDH knockdown blocked sarcosine-to-glycine conversion, we inferred that this shift in metabolite levels might impact other mitochondrial processes, particularly the glycine N-methyltransferase (GNMT)-catalyzed reaction, which converts glycine back to sarcosine [44, 45]. This reaction consumes S-adenosylmethionine (SAM) and consequently produces S-adenosylhomocysteine (SAH) [46–48]. We hypothesized that an elevated sarcosine-to-glycine ratio inhibited the GNMT-catalyzed reaction, leading to an accumulation of SAM (Fig. 5E). As expected, we observed increased sarcosine-to-glycine ratios and SAM-to-SAH ratios in SARDH-knockdown T cells, whereas these ratios decreased in SARDH-overexpressing T cells (Fig. 5F, G; Supplementary Fig. S10D, E). These findings suggested that the altered sarcosine-to-glycine ratio affected the efficiency of GNMT-catalyzed reverse transformation, subsequently modulating SAM levels.

### SARDH modulates T-cell function via methylation-dependent NF-κB inhibition

**Fig. 6:**
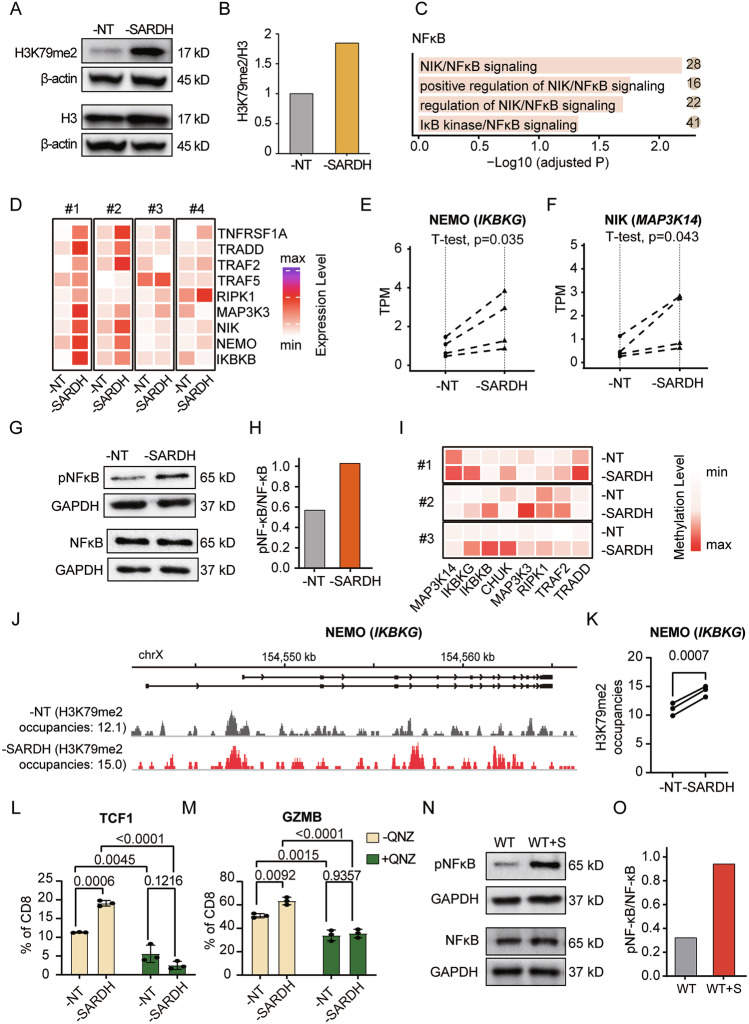
SARDH modulates T-cell function via methylation-dependent NF-κB inhibition. **A** Western blot analysis of H3 and H3K79me2 levels in human T cells. **B** Bar plot showing the densitometric analysis, which was used to quantify H3K79me2 levels. **C** Bar plot showing the NF-κB-related GO terms enriched in the SARDH-knockdown samples compared with the normal samples. The numbers in the solid circles indicate gene counts matched to the corresponding terms. **D** Heatmaps showing the expression levels of genes involved in NF-κB activation in four groups of nontargeting control (-NT) and SARDH knockdown (-SARDH) human CD8^+^ T cells. **E**–**F** Scatterplots showing the expression levels of *MAP3K14* (NIK) (**E**) and *IKBKG* (NEMO) (**F**) in nontargeting control and SARDH-knockdown human CD8^+^ T cells. The significance presented was calculated via a paired one-sided *t* test. *n* = 4. **G** Western blot analysis of p65 and phosphorylated p65 (Ser536) levels in human T cells (12 days after cell activation). **H** Bar plot showing the densitometric analysis, which was used to quantify NF-κB activation (phosphorylation levels of p65). **I** Heatmaps showing H3K79me2 enrichment within several genes involved in NF-κB activation across four groups of nontargeting control (-NT) and SARDH knockdown (-SARDH) conditions. **J** Genome browser view of the *IKBKG* gene region via IGV. **K** Scatterplot showing the quantification of H3K79me2 occupancy within the *IKBKG* gene. The significance presented was calculated via a paired one-sided *t* test. *n* = 3. **L** Bar plot illustrating the percentages of human CD8^+^ T cells expressing TCF-1 in the nontargeting control and *SARDH* knockdown groups with or without the addition of QNZ. *n* = 3. **M** Bar plot depicting the percentages of human CD8^+^ T cells expressing granzyme B in the nontargeting control and SARDH knockdown groups with or without the addition of QNZ. *n* = 3. In (**L**, **M**), the data are presented as the means with SDs, and all significance values were calculated via an unpaired *t* test. **N** Western blot analysis of p65 and phosphorylated p65 (Ser536) levels in human T cells treated (WT + S) or not treated (WT) with sarcosine (12 days after cell activation). **O** Bar plot showing the densitometric analysis, which was used to quantify NF-κB activation. All significances presented in the bar plots were calculated via an unpaired *t* test. All the bar plot data are presented as the means with SDs. All the data shown are representative of at least three independent experiments

As a well-established methyl donor, SAM is essential for DNA, RNA, and histone methylation. Fluctuations in SAM can influence gene expression through epigenetic modifications [49, 50]. For example, previous reports indicate that decreased SAM levels lead to diminished H3K79me2 in CD8^+^ T cells [51]. We measured H3K79me2 modification levels in T cells and found that these levels increased upon SARDH knockdown (Fig. 6A, B).

Given the multifaceted effects of epigenetic methylation, we conducted a GO enrichment analysis on the transcriptome instead of directly assessing all potential modifications, which provided an indirect yet comprehensive view of methylation-regulated gene expression and allowed us to identify SARDH-influenced pathways regulating T-cell properties. This analysis revealed an increase in NF-κB signaling upon SARDH knockdown (Fig. 6C). Additionally, at the transcriptional level, we observed increased expression of multiple genes involved in NF-κB activation. Notably, *IκB kinase-γ* (*IKBKG*, encoding NEMO), the regulatory subunit in the IKK complex necessary for canonical NF-κB activation, and *mitogen-activated protein kinase 14* (*MAP3K14*, encoding NIK), the upstream kinase in the noncanonical NF-κB activation pathway [52–54], were both upregulated (Fig. 6D–F).

To further validate these findings, we detected the phosphorylation of p65, a key indicator of NF-κB activation [55, 56]. The level of p65 phosphorylation significantly increased following SARDH knockdown and decreased with SARDH overexpression (Fig. 6G, H; Supplementary Fig. S11A, B). These findings indicated enhanced NF-κB signaling in SARDH-knockdown T cells. In parallel, we detected the activities of other pathways associated with T-cell activation. Upon SARDH knockdown, the AP-1 signaling pathway was upregulated, whereas the activity of the NFAT pathway was not significantly changed (Supplementary Fig. S11C, D).

To confirm that the NF-κB signaling pathway was affected by epigenetic methylation upon SARDH knockdown, we measured the H3K79me2 modification levels of genes associated with NF-κB activation, which has been reported to facilitate transcriptional elongation [57]. We detected elevated H3K79me2 on *IKBKG*, *MAP3K14*, and several other genes involved in NF-κB activation, which were also upregulated in our transcriptional analysis (Fig. 6I–K; Supplementary Fig. S11E, F). This finding was correlated with enhanced NF-κB activity following SARDH knockdown, providing support for the methylation-dependent regulatory mechanism of SARDH.

The role of the NF-κB signaling pathway in regulating various T-cell properties, such as activation, proliferation, differentiation, migration, and effector functions, has been well documented [55, 58–60]. On the basis of these findings, we hypothesized that SARDH influences T-cell properties, at least in part, via the NF-κB signaling pathway. To test this hypothesis, we treated CD8^+^ T cells with the NF-κB inhibitor QNZ to determine whether it could mitigate the effects of SARDH knockdown. As expected, QNZ effectively counteracted the improvements in stemness, cytotoxicity, and activation—indicated by increased expression ratios of TCF1, granzyme B, and CD69, respectively—which were attributed to reduced SARDH expression in CD8^+^ T cells (Fig. 6L, M; Supplementary Fig. S11G–J). These results suggested that SARDH could accelerate T-cell dysfunction by inhibiting the NF-κB signaling pathway.

Moreover, T cells cultured in high-sarcosine medium presented increased p65 phosphorylation (Fig. 6N, O), indicating that NF-κB signaling was elevated under conditions of sarcosine accumulation, which occurred following SARDH knockdown. These findings collectively implied that SARDH influences T-cell properties by modulating the relative concentration of sarcosine, which subsequently affects the levels of the methyl donor SAM. This modulation may alter methylation patterns and gene expression, including that of regulators of NF-κB signaling, thereby impacting cellular functions.

### Sarcosine accumulation induces T-cell expression of *SARDH*

**Fig. 7:**
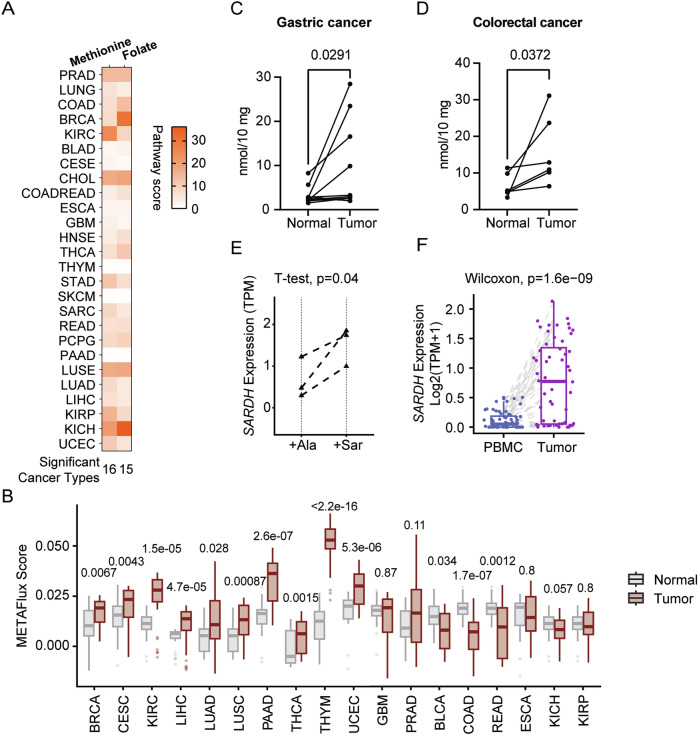
Sarcosine accumulation induces T-cell expression of *SARDH.* **A** Heatmap presenting the pathway scores of the methionine cycle and folate one-carbon metabolism in multiple cancer types [32]. **B** Boxplot showing the METAFlux score in tumor and normal tissues. All significances presented were calculated via Wilcoxon’s test. *n* = 30. **C**–**D** Scatterplots showing the concentration of sarcosine in tumors and corresponding normal tissues from gastric cancer (**C**) or colorectal cancer (**D**) patients. The significance presented was calculated via a paired one-sided *t* test. Gastric cancer: *n* = 10; colorectal cancer: *n* = 6. **E** Scatterplot showing the expression levels of *SARDH* in samples treated with alanine or sarcosine. The significance presented was calculated via a paired one-sided *t* test. *n* = 3. **F** Boxplot showing the *SARDH* expression levels in T cells from patient-derived peripheral blood samples and tumor tissues. The significance was calculated via Wilcoxon’s test

Investigating the impact of distinct metabolic pathways within the tumor microenvironment on infiltrating T cells is important for understanding tumor-driven immunosuppression. Specifically, we aimed to determine why *SARDH* is enriched in exhausted T-cell subtypes and whether its upregulation is associated with metabolic features within tumors. We hypothesized that sarcosine, as the substrate of SARDH, might influence SARDH expression on the basis of the concept of feedback regulation. We first examined the alterations in metabolic pathways responsible for sarcosine production, including the methionine cycle and folate one-carbon metabolism, across various cancer types [30, 61] to analyze the changes in sarcosine concentrations within tumors. Transcriptome comparisons between tumors and corresponding healthy tissues revealed significant dysregulation of these pathways in various cancers [32] (Fig. 7A). Enzymes involved in these pathways, such as AHCY, MAT1A, and SHMT2, were generally upregulated at the transcriptional level, suggesting increased sarcosine synthesis (Supplementary Fig. S12A, B). We subsequently utilized transcriptome data to predict the metabolic flux of sarcosine and found that multiple types of tumor tissues presented a stronger trend of excreting sarcosine than did the corresponding healthy tissue [10, 62] (Fig. 7B). These results implied that 1-C metabolism was active in tumors across multiple cancer types, potentially leading to the accumulation of sarcosine in the TME.

To validate this, we compared sarcosine concentrations in samples obtained from tumors and adjacent normal tissues in patients with gastric or colorectal cancer. In most cases, the sarcosine concentration was greater in tumors than in corresponding normal tissues (Fig. 7C, D). This observation aligns with several previous studies. For example, sarcosine has been detected in the urine of prostate cancer patients, where its presence is rare in healthy individuals, and its concentration has been correlated with prostate cancer progression [50, 63]. Moreover, higher sarcosine levels have been observed in the serum of gastric cancer patients than in that of healthy individuals [64]. These results support our hypothesis that sarcosine tends to accumulate inside tumors.

In vitro experiments revealed that treating T cells with sarcosine could upregulate *SARDH* expression, suggesting a negative feedback mechanism to maintain sarcosine homeostasis (Fig. 7E). In vivo, we also observed that T cells infiltrating tumors, where sarcosine was inferred to accumulate, presented higher *SARDH* expression than did T cells from patients’ peripheral blood (Fig. 7F). These findings suggest that T cells in the TME potentially upregulate *SARDH* in response to sarcosine enrichment (Supplementary Fig. S12C). The feedback mechanism exacerbates T-cell dysfunction in the TME to some extent.

## Discussion

In this study, we comprehensively analyzed the reprogrammed metabolic pathways and mitochondrial genes associated with T-cell exhaustion within the TME, leveraging the T-cell atlas depicted by pancancer single-cell sequencing. Our results underscore the dysregulation of 1-C metabolism within tumors and the detrimental impact of SARDH on T-cell fate and functions, suggesting new avenues for therapeutic interventions in cancer treatment.

The importance of SARDH, and more extensively, 1-C metabolism in tumor-infiltrating T cells, was previously underappreciated. In addition to SARDH, methylenetetrahydrofolate dehydrogenase 2 (MTHFD2), another enzyme involved in folate metabolism and previously linked to SAM production [65, 66], was also enriched in exhausted CD8^+^ T cells across all the cancer types we studied (Supplementary Fig. S1G), further indicating the potential link between altered 1-C metabolism and T-cell exhaustion. Notably, although we found that SARDH affects multiple aspects of T-cell fate and function, our preliminary data do not support a role for SARDH in modulating T-cell exhaustion, perhaps due to limitations in current detection strategies.

We therefore propose a model illustrating how SARDH impairs T-cell functions. In the TME, SARDH is upregulated to promote sarcosine consumption, enhancing GNMT-mediated glycine-to-sarcosine transformation. This process consumes the methyl donor SAM, thereby influencing epigenetic methylation. This, in turn, could alter the expression of multiple genes, impacting NF-κB signaling and other cellular functions. As reported, disrupted methionine metabolism in CD8^+^ T cells decreases SAM levels, resulting in diminished H3K79me2, lower STAT5 expression, and impaired T-cell immunity [51]. We also observed an increase in H3K79me2 occupancy in several key NF-κB-activating genes. These findings support our hypothesis that SARDH impedes T-cell functionality by compromising SAM-related epigenetic methylation.

However, our study has several limitations that should be acknowledged. First, the analysis of metabolism was primarily based on transcriptome data. A more direct assessment of the metabolome, especially that of sarcosine and other metabolites associated with SARDH, in tumors and infiltrating T cells would offer deeper insights. Identifying the cell types within the TME that secrete sarcosine would contribute to a better understanding of the regulation of SARDH expression. In addition, further investigations into the impact of the SARDH are still needed. For example, exploring mitochondrial functions and metabolic pathways, particularly 1-C metabolism in T cells, could provide a more profound understanding of its role and underlying mechanisms. Furthermore, the absence of validation for GNMT-related mechanisms and the lack of a more comprehensive analysis of SAM-mediated methylation beyond H3K79me2 indicate a gap in the understanding of the impact of SARDH on the NF-κB pathway and T-cell properties.

Given that SARDH has detrimental effects on T-cell effectiveness and exacerbates tumor malignancy, targeting SARDH as a metabolic checkpoint in T cells could be a promising anticancer strategy. To effectively implement this strategy, it is necessary to understand the mechanisms underlying this immune escape process, which prevents T cells from eliminating cancer cells. The scarcity of nutrients within tumors necessitates metabolic efficiency for survival, forcing various metabolic processes to adapt under the stress of nutrient and energy limitations. As the predominant cellular component within tumors, cancer cells exhibit increased proliferative and mutational capacities, likely outcompeting the ability of tumor-infiltrating T cells to adapt to the metabolic landscape of the tumor.

Previous reports have indicated that cancer cells have a greater ability to take up methionine—a crucial metabolite involved in 1-C metabolism and sarcosine synthesis—resulting in decreased methionine availability for cytotoxic T cells [51]. Our transcriptomic analysis across various cancers suggested that sarcosine synthesis is more active within tumors. Thus, cancer cells may prioritize the acquisition of materials involved in 1-C metabolism, such as methionine, over tumor-infiltrating T cells, leading to excessive sarcosine production. Excessive sarcosine may diffuse into the TME and be absorbed by tumor-infiltrating T cells, which adapt to the use of byproducts generated by cancer cells due to insufficient nutrient availability [13, 14]. The observed upregulation of *SARDH* upon sarcosine exposure in T cells suggests a mechanism aimed at optimizing sarcosine utilization by T cells. Overall, these adaptations may represent strategic shifts for more efficient resource and energy utilization, suggesting metabolism-targeted therapeutic interventions in cancer treatment.

## Materials and methods

### Metabolic analysis of tumor-infiltrating T cells

Pathway scores were calculated following the methods of Rosario et al. with the differentially expressed genes (DEGs) between T cells isolated from tumors and the corresponding normal tissue [32]. The data were collected from Zheng et al. [10]. The DEGs were calculated via the “FindMarker” function in “Seurat” with the Benjamini‒Hochberg approach for multiple hypothesis correction [67].

### Computational pipeline to prioritize mitochondria-related genes associated with CD8^+^ T-cell exhaustion

1. Mitochondria-related genes: We collected mitochondria-related genes from MitoCarta [36], MitoProteome [37], and AmiGO [38] and excluded genes present in only one gene set. Finally, a total of 1264 genes were used as mitochondria-related genes in this study.
2. Single-cell RNA-seq data processing: Single-cell transcriptomic data of T cells were collected from Zheng et al. with well-defined cell annotations10. The cells from four exhausted CD8^+^ T-cell subtypes, including GZMK^+^ Tex, TCF7^+^ Tex, OXPHOX^-^ Tex, and terminal Tex, were merged as exhausted CD8^+^ T cells. Cancer types with fewer than 100 exhausted CD8^+^ T cells were removed from the analysis. DEGs were identified as previously mentioned. Genes with an adjusted *p* value less than 0.05 were considered significantly differentially expressed. The log_2_(fold change) (log2FC) for each gene was calculated by subtracting the log_2_-transformed mean count in exhausted and nonexhausted cells. To evaluate each gene in all cancer types, we used the “sumlog” function from the “metap” package for meta-analysis to combine *p* values detected from different cancer types [68]. Additionally, we calculated the median log2FC value across different cancer types for each gene. Finally, we obtained a list of differentially expressed genes with log2FC and *p* values.
3. Enrichment score and exhaustion score: For each gene, we defined its enrichment score according to the descending ranking of its log2FC values. For genes significantly upregulated in exhausted CD8^+^ T cells, we further performed Pearson’s correlation analysis between gene expression levels and the signature scores of T-cell exhaustion in CD8^+^ T cells [10], which were defined on the basis of the average expression levels of *PDCD1*, *CTLA4*, *HAVCR2*, *LAG3*, and *TOX*. *P* values were adjusted via the Benjamini‒Hochberg approach. We defined the exhaustion score of each gene according to the descending ranking of correlation coefficients.

### RNA sequencing

The sequencing libraries were constructed via the TruePrep RNA Library Prep Kit for Illumina (Vazyme), with a single-cell cDNA quality check via the ChamQ SYBR qPCR Master Mix (Vazyme). The samples were subsequently sequenced via the Illumina Nova PE150 platform.

### Sequencing data processing and identification of differentially expressed genes

The RNA-seq data were first processed to filter out low-quality reads via fastp [69], with (1) “N” bases accounting for 3% of the read length, (2) bases with quality <3 accounting for 50% of the read length, (3) containing adapter sequences, (4) >40% of the bases unqualified with phred quality <Q15, or (5) having an average quality score <average quality. Kallisto [70] was subsequently used to quantify the abundance of transcripts. To summarize transcript-level abundance estimates for gene-level analysis, the tximport package [71] from R bioconductor was used with the parameter “countsFromAbundance = lengthScaledTPM” to correct library size and average transcript length across samples. Differential gene expression analysis in paired samples was performed by using the DESeq2 package [72] from the R bioconductor. Only the genes with adjusted *p* values less than 0.05 were considered to be differentially expressed.

### Gene expression specificity analysis

Single-cell transcriptomic data from The Human Protein Atlas were collected to detect whether the genes were widely expressed in various cell types in healthy human tissue [39]. For each candidate gene, we set the 10% of the maximum transcripts per million (TPM) value among all 81 collected cell types as a threshold and subsequently defined the number of cell types whose TPM exceeded the threshold.

To confirm the expression specificity of the candidate genes in T cells, we determined the rank of each gene’s expression level in T cells among all collected cell types on the basis of the TPM values. We subsequently calculated the ratio of its expression level in T cells to the median TPM in other cell types.

### Gene set enrichment analysis

The clusterProfiler from the Bioconductor package was used to perform gene set enrichment analysis [73]. Briefly, for functional enrichment analysis, all DEGs were mapped to terms in the Gene Ontology (GO) database, and significantly enriched GO terms were identified via an adjusted *p* value threshold of less than 0.05. GO term analysis classified genes into three subgroups, namely, biological process (BP), cellular component (CC) and molecular function (MF). Only BP GO terms were considered in this study.

### Human T-cellcell isolation and culture

HLA-A* 0201 peripheral blood mononuclear cells (PBMCs) were separated from human blood via Histopaque®-1077 (Merck). CD3^+^ T cells were subsequently sorted from human PBMCs via the EasySep™ Human CD3^+^ T-Cell Isolation Kit (STEMCELL Technologies). The sorted T cells were cultured in HIPP-T009 medium (BioEngine) supplemented with 5% FBS (Thermo Fisher Scientific), 10 ng/μL IL-2, IL-7, and IL-15 (PEPROTECH) at 37 °C with 5% CO_2_. CD8^+^ T cells were activated via Dynabeads™ Human T-Activator CD3/CD28 for T-cell expansion and activation (Thermo Fisher Scientific) at an equivalent number to that of CD3^+^ T cells. The beads were removed on day 4.

### Lentivirus packaging and transfection

A lentivirus packaging system consisting of pVSVG and pR8.74 was utilized for DNA transfection into Lenti-X 293T packaging cell lines to produce target-sequence-containing viruses via X-tremeGENE™ HP DNA Transfection Reagent (Merck), followed by a virus harvest after 2 days of cultivation [74]. T cells were then transfected with these viruses after stimulation for 2 days.

### Knocking down SARDH in human CD8^+^ T cells

SARDH was knocked down in human T cells via siRNA or shRNA transfection. siRNAs were transfected via Entranster^TM^-R4000 (Engreen Biosystems), and shRNAs were constitutively transfected with lentiviruses.

### RNA expression analysis

The cells were harvested 2 days after siRNA transfection. Total RNA was isolated with a Quick-RNA™ Microprep Kit (Zymo Research). To quantify the expression level of SARDH mRNA, we conducted reverse transcription and qPCR using HiScript® III RT SuperMix for qPCR (Vazyme) and ChamQ SYBR® qPCR Master Mix (Vazyme), respectively. The expression of SARDH mRNA was normalized to that of the housekeeping gene GAPDH. Each sample was subjected to duplicate runs, with two nontemplate control wells included in each PCR experiment.

The sequences of primers used were as follows: SARDH-F (5’-CGCATCCAGGGCATTCAGAAC), SARDH-R (5’-AAGATGGGGTTGGCCTCATAG), GAPDH-F (5’-ACAACTTTGGTATCGTGGAAGG), and GAPDH-R (5’-GCCATCACGCCACAGTTTC).

### Western blot

Western blotting was used to detect the SARDH level; the phosphorylation levels of p65, c-Jun and p38; and the H3K79me2 level in T cells. The cells were harvested and resuspended in RIPA buffer (Thermo Fisher Scientific) supplemented with protease and phosphatase inhibitor cocktail for general use (Beyotime). The lysates were incubated on ice for 15 min, followed by centrifugation at 14,000 × *g* for 5 min at 4 °C, after which the supernatants were collected. Equal amounts of protein, quantified via the Enhanced BCA Protein Assay Kit (Beyotime) and the Sunrise absorbance microplate reader (Tecan), were mixed with 5x SDS‒PAGE sample loading buffer (Beyotime) and boiled at 95 °C for 5 min. The samples were then loaded onto 8% SDS‒PAGE gels (Cwbiotech) and transferred to a PVDF membrane via a Trans-blot semidry transfer cell (Thermo Fisher Scientific). After the membranes were blocked (Beyotime), they were incubated with the appropriate primary antibodies, followed by incubation with an HRP-conjugated secondary antibody. The signals were visualized via SuperSignal™ West Pico PLUS (Thermo Fisher Scientific) on PVDF membranes.

The following antibodies were used: rabbit polyclonal anti-SARDH (1:250), rabbit monoclonal anti-NF-κB (1:500), rabbit monoclonal anti-p-NF-κB (1:250), rabbit monoclonal anti-c-Jun (1:500), rabbit monoclonal anti-p-c-Jun (1:500), rabbit monoclonal anti-p38-MAPK (1:500), rabbit monoclonal anti-p-p38-MAPK (1:500), rabbit monoclonal anti-H3K79me2 (1:500), rabbit monoclonal anti-H3 (1:1000), rabbit monoclonal anti-GAPDH (1:2000), rabbit monoclonal anti-β-actin (1:3000), and goat polyclonal anti-rabbit IgG-HRP (1:5000) antibodies.

### Detection of mitochondrial membrane potential

T cells were labeled with 100 nM tetramethylrhodamine ethyl ester (TMRE; Thermo Fisher Scientific) for 30 min at 37 °C. The geometric mean fluorescence intensity was detected via flow cytometry.

### Flow cytometry antibody staining

To eliminate interference from dead cells in the experiment, the Zombie NIR™ Fixable Viability Kit (BioLegend) was used to distinguish live cells from dead cells. For extracellular staining, the cells were washed with FACS buffer (PBS containing 2% FBS), followed by the addition of antibodies and incubation at 4 °C for 60 min. For intracellular staining, the cells were washed with FACS buffer and then fixed at 4 °C with 4% fixative solution (Solarbio). The cells were gently permeabilized with methanol. For human cells, BD Pharmingen™ Human BD Fc Block™ (BD Biosciences) was used to prevent nonspecific binding. For murine cells, purified rat anti-mouse CD16/CD32 (BD Biosciences) was utilized. After incubation at room temperature for 15 min, antibodies were added, followed by a 1-hour incubation at 4 °C. For data acquisition and analysis, LSRFortessa (BD Biosciences) and FlowJo (BD Biosciences) were used.

### Flow cytometry analysis of human T-cell properties and functions

Human T cells were cultured in vitro for the detection of CD69 on day 1; TCF-1, CCR7, and CD45RA on days 10–14; and exhaustion markers, including PD-1, Tim-3, and Lag-3, on day 20 postactivation.

### Construction of the human T-cell exhaustion model

HLA-A*0201 PBMCs were isolated and cultured as described above. CD8^+^ T cells were activated via Dynabeads™ Human T-Activator CD3/CD28 for T-cell expansion and activation (Thermo Fisher Scientific) and were added at a 1:1 ratio to CD3^+^ T cells. To induce T-cell exhaustion, activation was performed every two days for a total of four stimulations. Exhaustion marker expression was assessed approximately 20 days after initial activation.

### Cell proliferation analysis

For cell staining, human T cells transfected with siRNA were incubated in PBS (Thermo Fisher Scientific) supplemented with 5 μM CFSE (Thermo Fisher Scientific) for 5 min at 37 °C and then washed with PBS before being resuspended in fresh culture medium. AccuCheck Counting Beads (Thermo Fisher Scientific) were uniformly introduced into the cell suspension for relative quantification. The stained cells were cultured under the standard conditions mentioned above and subjected to flow cytometry analysis on day 3 postactivation to obtain distributions of cell fluorescence intensity at 488 nm. A threshold was subsequently set to determine cell proliferation, and the numbers of proliferated cells were normalized to those of counting beads.

### In vitro T-cell killing analysis

We constructed MART-1-specific CD8^+^ T-cell clones by transfecting vectors containing specific TCR α- and β-chains (DMF5) into CD8^+^ T cells through lentiviruses [75–77] on day 1. The culture conditions were as described above.

Killing analyses were performed 12 days after cell activation. Both MART-1^+^ A375 and T2 cells were utilized as target cells [78]. MART-1^+^ A375 cells were harvested at a concentration of 5 × 10^5^ cells/mL with HIPP-T009 medium. T2 cells were harvested at the same concentration in IMDM (without FBS) (Thermo Fisher Scientific). The samples were subsequently incubated overnight with 10 μg/mL peptide (LAGIGILTV) to facilitate loading onto their surface, followed by the replacement of the medium with HIPP-T009.

For relative quantification, AccuCheck Counting Beads (Thermo Fisher Scientific) were uniformly introduced into the target cell suspension. The TCR-T and target cells were then combined in 96-well plates at a 1:1 ratio and incubated for a minimum of 6 h. Flow cytometry was subsequently employed to determine the killing efficiency, followed by calculations utilizing the following formula: [1-([TC]_e_/[beads]_e_)/([TC]_c_/[beads]_c_)] ×100%. [TC]_e_ and [beads]_e_ represent the numbers of target cells and beads, respectively, in the experimental sample, whereas [TC]_c_ and [beads]_c_ denote those in the control sample, where effector cells are T cells lacking expression of the MART-1-specific TCR.

### Migration assays

Migration assays were performed 10–14 days postcell activation via a Tissue Culture Plate Insert (LABSELECT) with an 8 μm polycarbonate membrane. Geltrex™ LDEV-Free Reduced Growth Factor Basement Membrane Matrix (Thermo Fisher Scientific) was diluted at a ratio of 1:7 and loaded in the upper chamber at a volume of 100 μL/well. After the Matrigel solidified, T cells were added to the wells and allowed to transmigrate at 37 °C with 5% CO_2_. Simultaneously, the counting beads mentioned above were uniformly introduced into the wells. The migrated cells were collected for relative quantification via flow cytometry; the relative number of migrated cells was standardized by comparing the ratio of the number of collected cells to the number of counting beads.

### In vitro murine CD8^+^ T-cellcell isolation and culture

Subiliac lymph nodes were dissected from the mice and dissociated mechanically in PBS. The cell suspensions were then filtered through a 70 μm cell strainer (Biosharp), centrifuged, and resuspended in EasySep buffer. Lymphoid CD8^+^ T cells were isolated via an EasySep™ Mouse CD8^+^ T-Cell Isolation Kit (STEMCELL Technologies). The isolated cells were then cultured in RPMI-1640 medium (Thermo Fisher Scientific) supplemented with 10% FBS at 37 °C with 5% CO_2_.

### Three-dimensional mouse tumor spheroid-T-cell coculture system

To generate spheroids, we seeded 3000 tdTomato^+^ MC38-OVA cells/well into a U-bottom 96-well plate treated with a decellularization agent. The cells were cultured in DMEM under standard conditions at 37 °C with 5% CO_2_. After ~48 h, spheroids were successfully formed. An equal number of CD8^+^ T cells were subsequently added to the wells and cocultured for 48 h.

### Human three-dimensional tumor organoid/spheroid-T-cell coculture system

For primary organoid generation, human colorectal cancer tumor tissue blocks were placed in 45 mL of ice-cold DMEM (Thermo Fisher Scientific) for collection. After fat or muscle tissues were removed, the tissues were cut into small pieces of 1–3 mm^3^ using surgical scissors or scalpels in a 10 cm cell culture dish. Colon digestion solution (DMEM + 500 U/mL collagenase IV (Thermo Fisher Scientific) + 1.5 mg/mL collagenase II (Thermo Fisher Scientific) + 20 mg/mL hyaluronidase (Merck) + 0.1 mg/mL dispase type II (Thermo Fisher Scientific) + 10 μM Y27632 (STEMCELL Technologies) + 1% FBS) was subsequently used for tissue digestion. When the mixture became cloudy and the remaining tissue fragments were broken, the mixture was pipetted 10–20 times to further promote tissue fragmentation. The cells were then washed with fresh medium and filtered through a 70 μm cell strainer. The filtered cells were centrifuged, and if the pellet was red in color, it was subjected to red blood cell lysis via ACK lysis buffer (Bingene) at 4 °C for 5 min. After lysis, the cells were washed with PBS in a 15 mL tube and then centrifuged again, followed by cell staining with CFSE as described above for cell proliferation analysis. The resulting cell pellet was resuspended and seeded in the 96-well plate mentioned above. The culture medium was replaced every 2‒3 days until organoids formed.

For EGFP^+^ A375 spheroids, cells were seeded at a concentration of 10,000 cells/well into the 96-well plate mentioned above and cultured in DMEM at 37 °C with 5% CO_2_. Spheroids formed after 48 h of culture.

For human T-cell staining, after 10–14 days, the cells were incubated in DPBS supplemented with 1 μM CM-DiI (Thermo Fisher Scientific) for 15 min at 37 °C and 15 min at 4 °C, washed with PBS and resuspended in fresh culture medium. Stained T cells were added to the wells (100,000 cells/well for primary spheroids and 10,000 cells/well for EGFP^+^ A375 spheroids) where the spheroids were cultured; T cells and spheroids were then cocultured for an additional 48 h.

### T-cell infiltration imaging and analysis

To investigate T-cell infiltration into tumor spheroids, we fixed the cocultured T-cell-spheroid system with 4% fixative solution after a 48-hour incubation period. The spheroids were subsequently subjected to layerwise imaging via either a laser scanning confocal microscope or a two-photon confocal microscope (Leica). The excitation of green fluorescence was achieved via a 488 nm laser, whereas a 552 nm laser was employed for red fluorescence. Three-dimensional reconstruction of the data obtained through LSCM/two-photon microscopy was conducted via the software Imaris, version 9.0.1. Spheroid boundaries were delineated on the basis of fluorescence signals via the “surfaces” module. Following this, the “spots” function was employed to quantify the number of T cells within the spheroid, with a spot diameter threshold set at 10 nm.

### Flow cytometry for mouse tumor spheroid-T-cell coculture samples

To generate spheroids, we seeded 10,000 tdTomato^+^ MC38-OVA cells/well via the method described above. The spheroids, along with the culture medium, were aspirated via a pipette and filtered through a 70 μm cell strainer. The spheroids were then washed with PBS. Moreover, the filtrate, along with the culture medium, contained noninfiltrating T cells. The spheroids on the filter were digested in 0.25% trypsin-EDTA until they had dissolved, after which they were gently pipetted, and the digestion was terminated with DMEM. The resulting mixture contained cancer cells and infiltrating T cells. Both samples were centrifuged and resuspended in 200 µL of PBS, and an equal number of counting beads were added for flow cytometry analysis.

For data acquisition and analysis, LSRFortessa (BD Biosciences) and FlowJo (BD Biosciences) were used. The number of infiltrating and noninfiltrating T cells in each sample was determined on the basis of the ratio of T cells to counting beads.

### 3D cytotoxicity imaging and analysis

To visualize and assess the cytotoxicity of T cells against tumor spheroids, the spheroids were transferred to a new U-bottom 96-well plate and suspended in 200 μL of PBS. One drop of NucRed^TM^ Dead 647 (Thermo Fisher Scientific) was subsequently added to each well, and the plate was incubated at 37 °C for 15 min. Imaging was conducted via an inverted fluorescence microscope (Leica) with a far-red channel to capture signals indicative of cell death. Image analysis, performed with ImageJ or Fiji, was used to quantify the area in pixel units.

### Mouse subcutaneous tumor model

All animal procedures were approved by the IACUC Committee of Peking University (Ethics approval ID: BIOPIC-ZhangZM-2). All the mice were housed in a pathogen-free facility and provided unrestricted access to water and chow. The mice were female and 8–12 weeks old at the time of implantation.

NSG mice (Cat. NO. NM-NSG-001) were purchased from Shanghai Model Organisms Center, Inc. Subcutaneous inoculation of MART-1^+^ A375 cells in a PBS/Matrigel (Corning) mixture was performed at a concentration of 1 × 10^5^ cells per NSG mouse. Tumor size was measured with calipers, and tumor volume was calculated via the formula V = 0.5 × a × b^2^ (a: major axis, b: minor axis). When the tumor volume reached approximately 50 mm^3^, 3 × 10^6^ MART-1-specific TCR-T cells were administered weekly via tail vein injections. The mice were humanely euthanized when the tumor size reached nearly 1500 mm^3^. OT-1^+^ mice were obtained from Cyagen Biosciences (Suzhou) Inc. For OT-1^+^ mice, subcutaneous inoculation and tumor size measurement were performed via the same procedure.

For cell sample preparation, tumors were dissected from the mice, cut into small pieces of 1–3 mm^3^ using surgical scissors or scalpels in gentleMACS™ C Tubes (Miltenyi Biotec) and subsequently digested via a tumor dissociation kit (Miltenyi Biotec). The cell suspensions were then filtered through a 70 μm cell strainer, washed with PBS, and resuspended in FACS buffer.

### Detection of the sarcosine concentration

The sarcosine concentration was determined via a Sarcosine Assay Kit (Merck). The samples were prepared by homogenizing 2 × 10^6 ^T cells in 100 µL of Sarcosine Assay Buffer, followed by centrifugation at 13,000 × *g* for 10 min to remove insoluble material. For the assay, 50 µL of Master Reaction Mix, consisting of 46 µL of Sarcosine Assay Buffer, 2 µL of Sarcosine Enzyme Mix, and 2 µL of Sarcosine Probe, was added to each well and gently mixed thoroughly. This mixture was incubated at 37 °C for 60 min in the dark, after which the absorbance was measured at 570 nm for colorimetric detection. Sarcosine concentrations in samples were determined by comparing their readings to the standard curve generated from the known concentrations of sarcosine standards.

### Liquid chromatography‒mass spectrometry (LC‒MS) to detect Sar/Gly and SAM/SAH

Frozen cell samples consisting of ~5 × 10^6 ^T cells were thawed at 4 °C, followed by the addition of 400 μL of 75% methanol. The extracts were then vortexed, ultrasonicated on ice for 15 min, and centrifuged at 17,000 × *g* for 15 min. The supernatants were collected for analysis. Chromatographic separation was performed on a ZORBAX Rx-C8 column (4.6 mm × 5 mm, 1.8 µm particle size) via binary gradient elution, with mobile phase A consisting of 200 mM 3-nitrophenylhydrazine hydrochloride in 75% methanol and mobile phase B consisting of 96 mM EDAC and 6% pyridine in methanol. The flow rate was set at 0.4 mL/min, and the injection volume was 1 µL. The mass spectrometer was operated in negative ion mode with an electrospray ionization (ESI) source. The quality control samples and calibration standards were analyzed alongside the experimental samples to ensure the reliability and reproducibility of the results.

### Detection of H3K79me2

For H3K79me2 detection, 50,000T cells per sample were collected and processed via CUT&Tag. The isolation of H3K79me2-modified DNA fragments and library construction were performed via the Hyperactive Universal CUT&Tag Assay Kit for Illumina Pro (Vazyme). Afterward, the libraries were multiplexed and sequenced on either an Illumina HiSeq/Novaseq or MGI2000 instrument via a 2 × 150 paired-end configuration. The raw data were subjected to quality control via Cutadapt (version 1.9.1) to remove technical sequences and low-quality bases and were normalized via DNA Spike-in [79]. The normalized and cleaned reads were then aligned to the reference genome GRCh38.109.

Methylation peaks were visualized via the Integrative Genomics Viewer (IGV) to assess signal distribution across regions of interest [80]. The quantification was performed via multiBigwigSummary from deepTools2 [81].

### Comparison of metabolism between tumor and corresponding normal tissues

Pathway scores of the methionine cycle and folate one-carbon metabolism were obtained from the Supplementary Data 3 provided by Rosario et al. [32].

Gene expression data for tumor and normal tissues were obtained from TCGA and GTEx, respectively. The RNA-seq expression data from TCGA were obtained via the TCGAbiolinks package [82]. GTEx samples, consisting of more than 9000 RNA-seq samples, were downloaded from the Genotype-Tissue Expression Project (https://gtexportal.org/home/). To address batch effects, a sample–gene expression matrix was created for each tumor–normal tissue pair by merging the gene expression levels of the corresponding TCGA and GTEx samples. The “normalize.quantiles” function from the “preprocessCore” package was utilized to correct for nonbiological variations [83]. Wilcoxon’s test was used to calculate the *p* value.

The “METAFlux” package was used to predict the metabolic flux of sarcosine in tumors and corresponding normal tissues with RNA expression data from the TCGA and GTEx [62] databases. Wilcoxon’s test was used to calculate the *p* value.

### Statistics for analysis of experimental data

All the statistical analyses were conducted via GraphPad Prism (GraphPad). Unless otherwise specified in the Fig. legend, the data are presented as the means with SDs and were analyzed via a two-tailed *t* test or ANOVA. Box plots illustrate the median with the 95th percentile, and error bars indicate the maximal deviation. Significance was determined at *p* ≤ 0.05, with * for *p* ≤ 0.05, ** for *p* ≤ 0.01, *** for *p* ≤ 0.001, and **** for *p* ≤ 0.0001.

## Supplementary information

Fig. S1 1-C metabolism is reprogrammed during tumor infiltration and SARDH is specifically enriched in exhausted T cells, related to Fig. 1

Fig. S2 Knocking down SARDH with RNAi, related to Fig. 2

Fig. S3 SARDH restricts T cell properties, related to Fig. 2

Fig. S4 SARDH restricts T cell cytotoxicity, related to Fig. 2

Fig. S5 SARDH influences T cell differentiation and proliferation, related to Fig. 2

Fig. S6 SARDH inhibits the migration and infiltration of T cells, related to Fig. 3

Fig. S7 SARDH impairs the tumor control ability of T cells in vivo, related to Fig. 4

Fig. S8. SARDH impairs the CD8+ T cell properties in vivo, related to Fig. 4

Fig. S9 SARDH influences CD8+ T cell differentiation in vivo, related to Fig. 4

Fig. S10 SARDH inhibits pathway-related cell properties by modulating the related metabolites, related to Fig. 5

Fig. S11 SARDH modulates T-cell function via methylation-dependent NF-κB inhibition, related to Fig. 6

Fig. S12 Dysregulation of 1-C metabolism in tumor, related to Fig. 7

Supplementary figure Legend
