# Supplementary figures and images for: SARDH in the 1-C metabolism sculpts the T-cell fate and serves as a potential cancer therapeutic target
Source: Cell Mol Immunol. 2025 Aug 20;22(11):1363–78. doi: 10.1038/s41423-025-01331-5 (PMC12575850; doi:10.1038/s41423-025-01331-5)

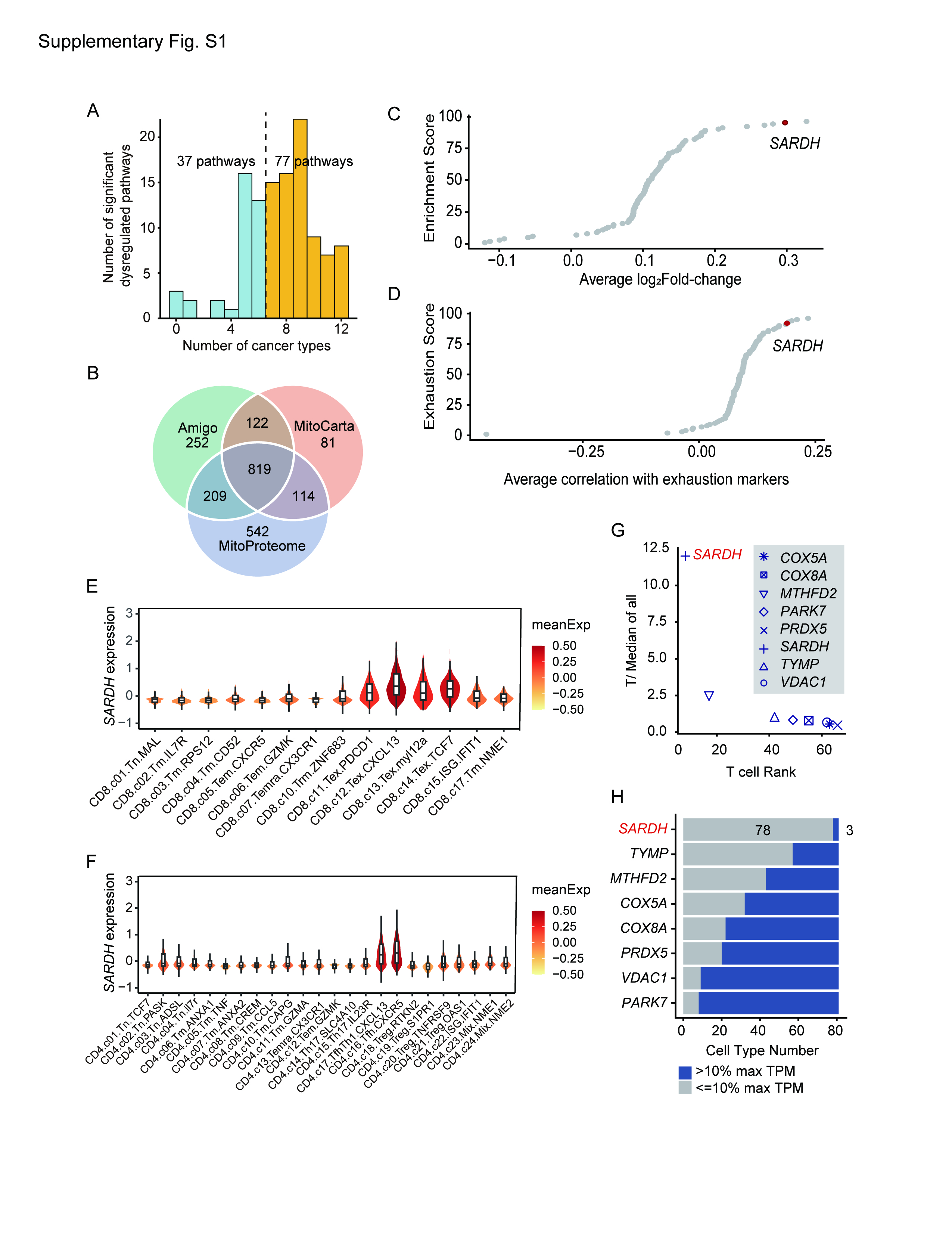

Supplement: Supplementary file 1 — Fig. S1 1-C metabolism is reprogrammed during tumor infiltration and SARDH is specifically enriched in exhausted T cells, related to Fig. 1 [file 41423_2025_1331_MOESM1_ESM.jpg]

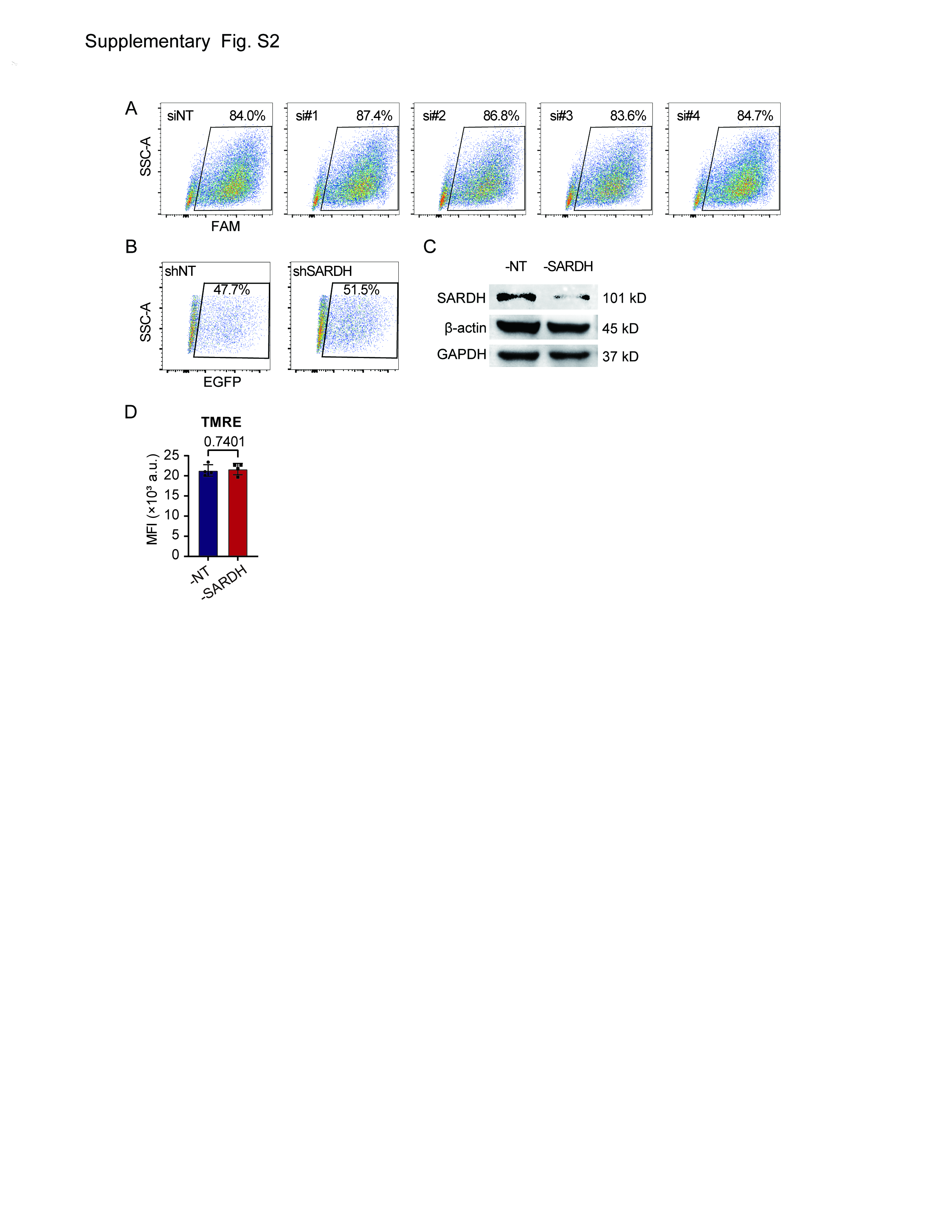

Supplement: Supplementary file 2 — Fig. S2 Knocking down SARDH with RNAi, related to Fig. 2 [file 41423_2025_1331_MOESM2_ESM.jpg]

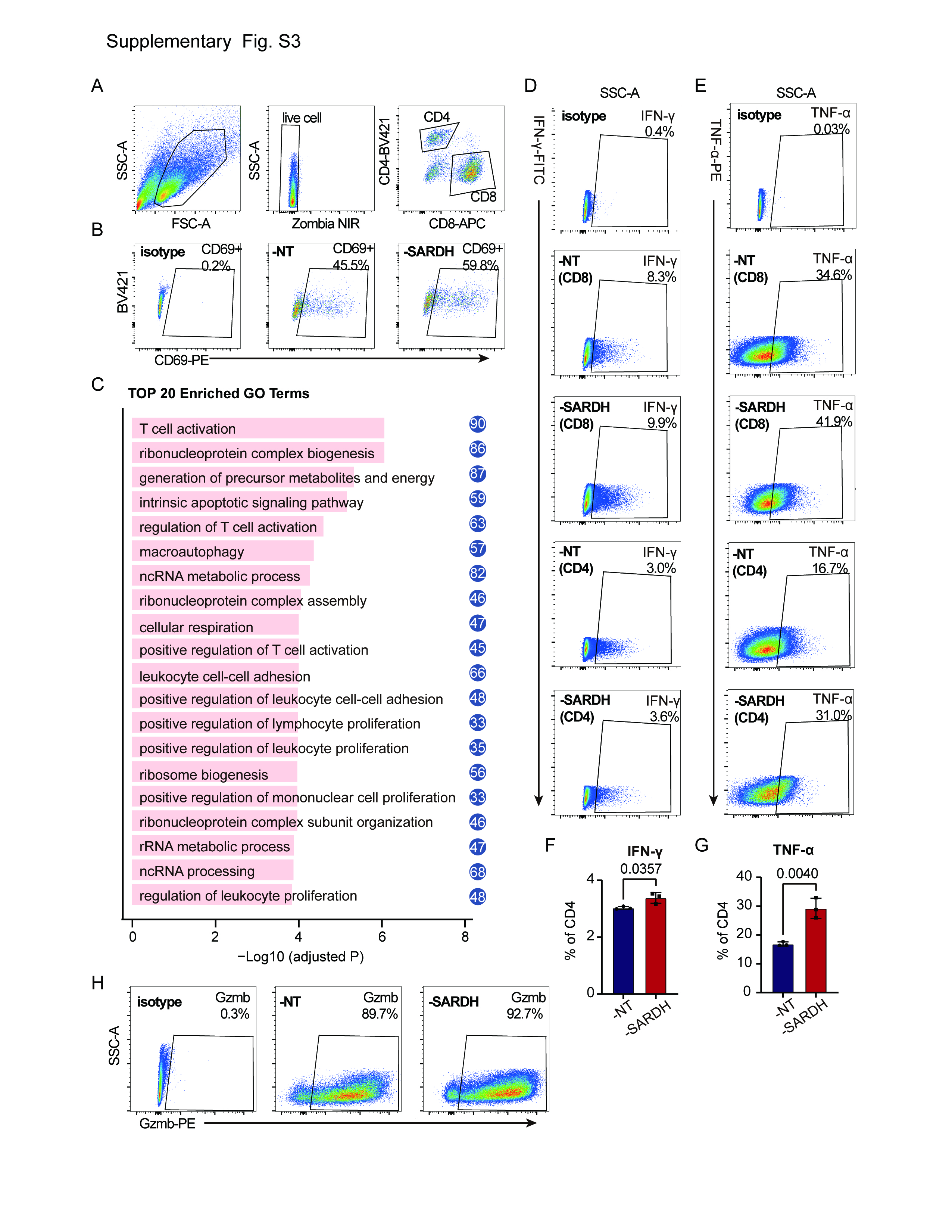

Supplement: Supplementary file 3 — Fig. S3 SARDH restricts T cell properties, related to Fig. 2 [file 41423_2025_1331_MOESM3_ESM.jpg]

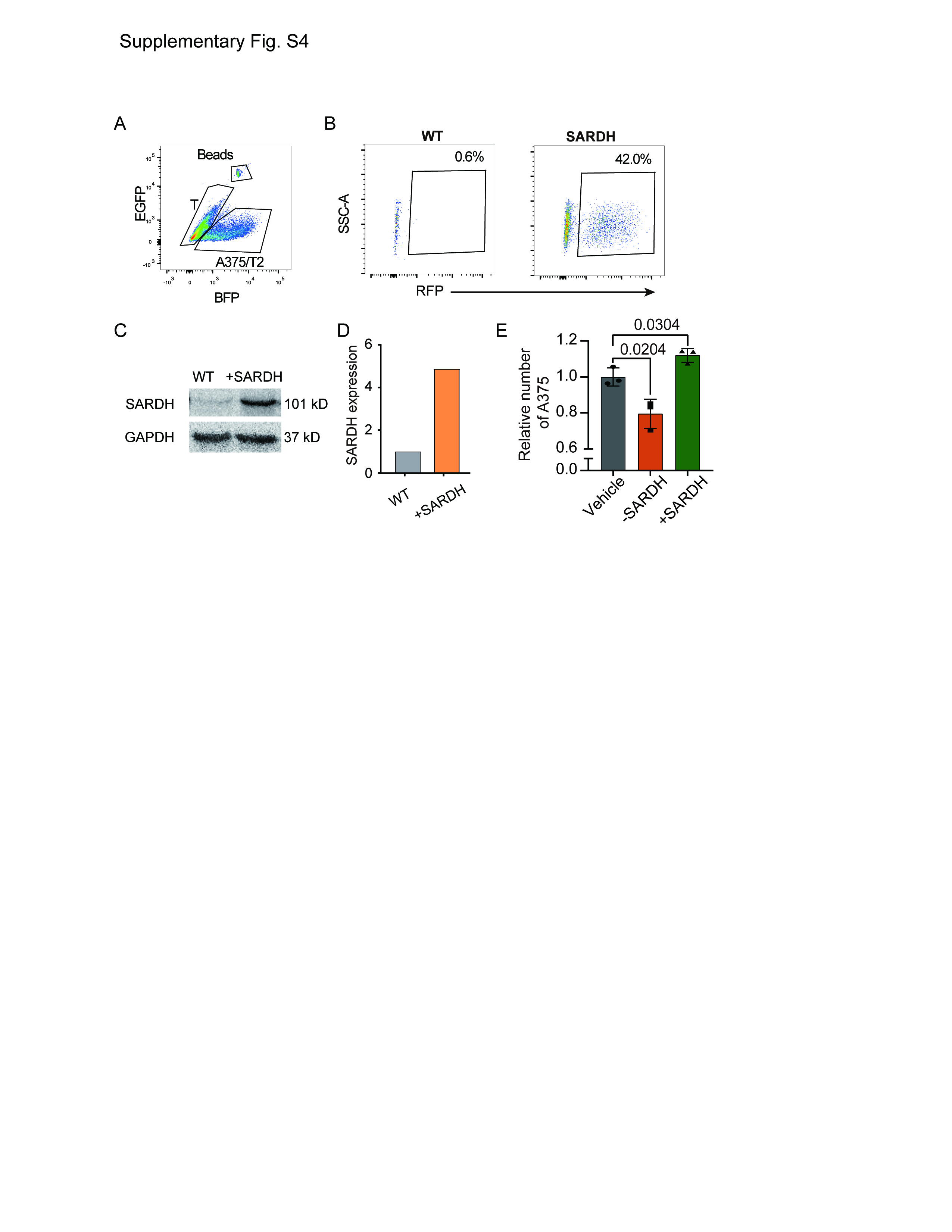

Supplement: Supplementary file 4 — Fig. S4 SARDH restricts T cell cytotoxicity, related to Fig. 2 [file 41423_2025_1331_MOESM4_ESM.jpg]

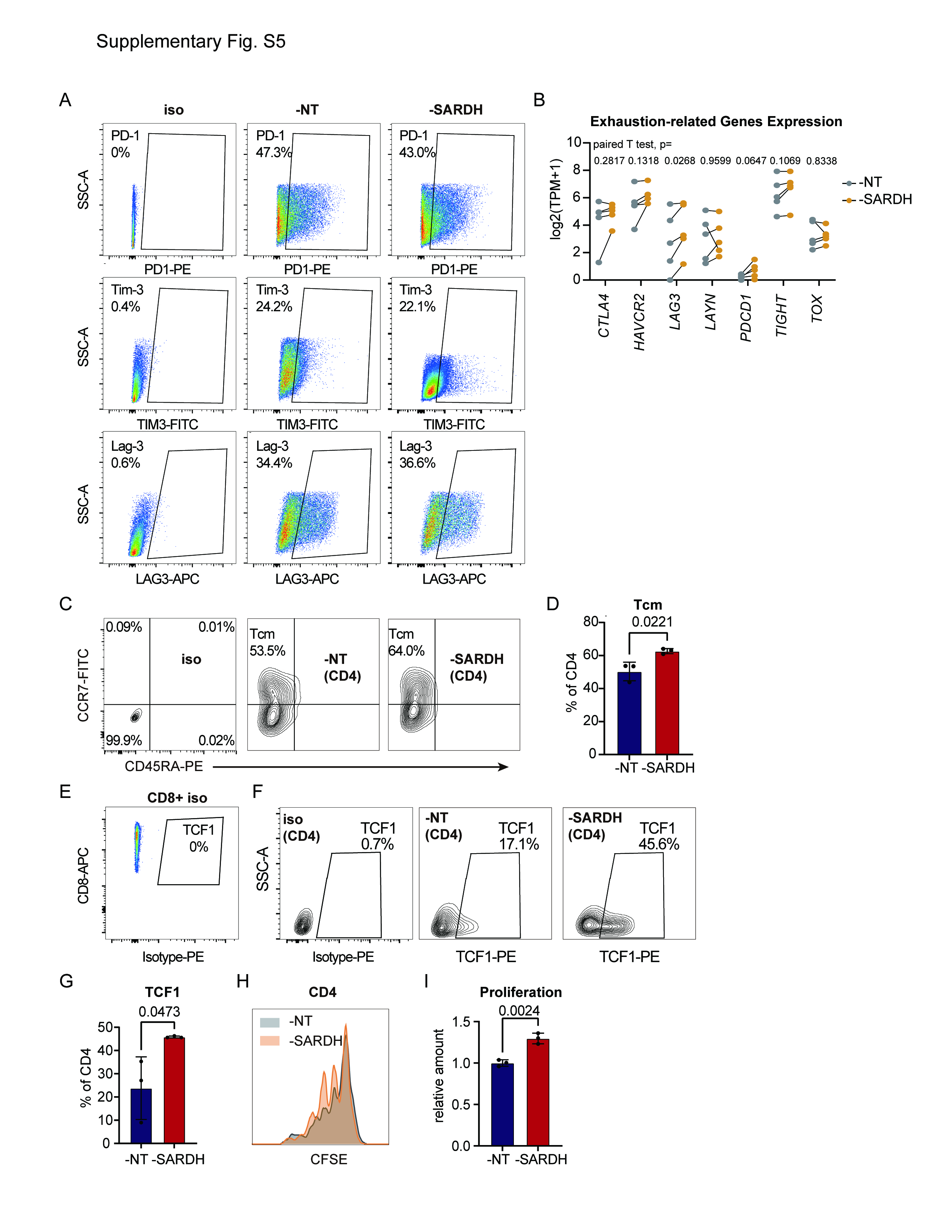

Supplement: Supplementary file 5 — Fig. S5 SARDH influences T cell differentiation and proliferation, related to Fig. 2 [file 41423_2025_1331_MOESM5_ESM.jpg]

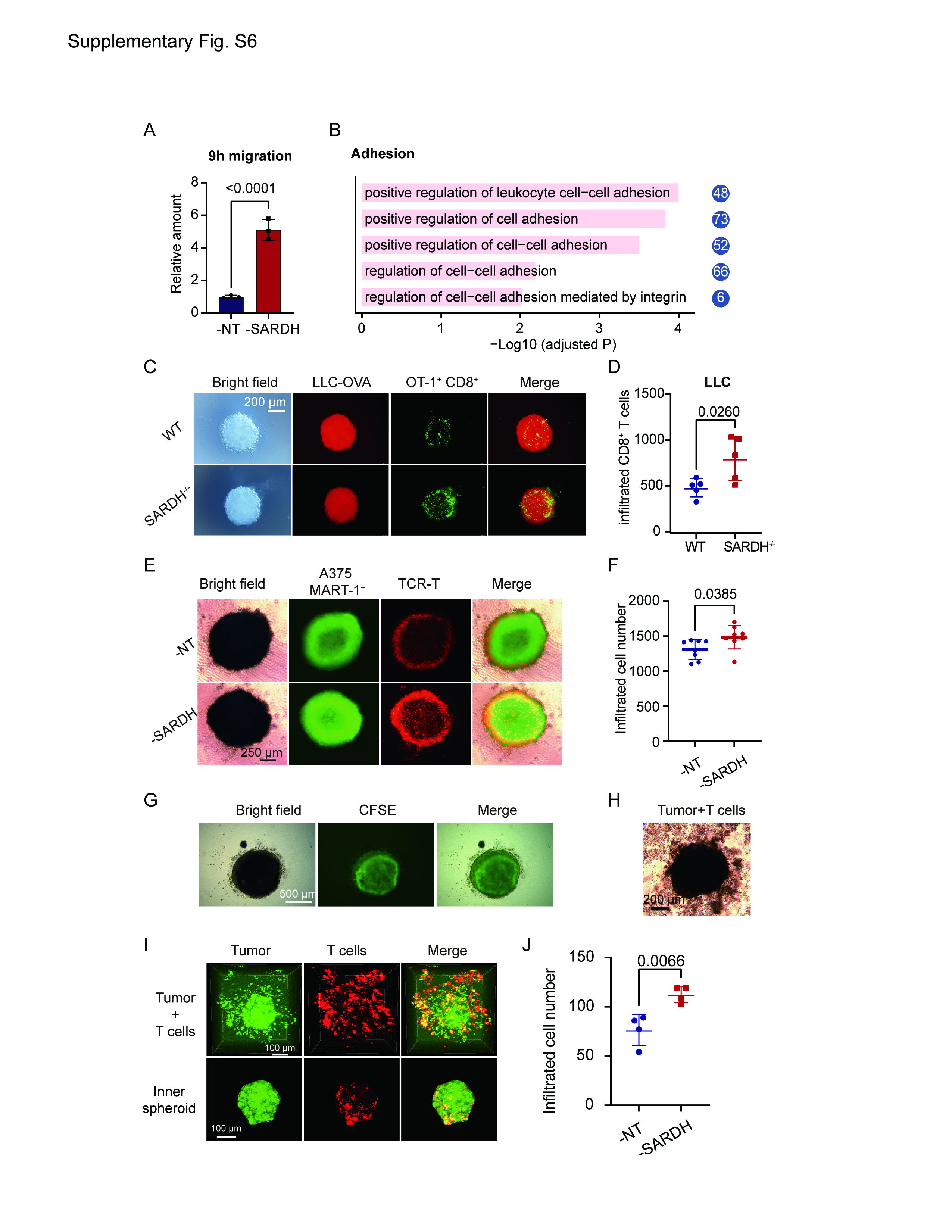

Supplement: Supplementary file 6 — Fig. S6 SARDH inhibits the migration and infiltration of T cells, related to Fig. 3 [file 41423_2025_1331_MOESM6_ESM.jpg]

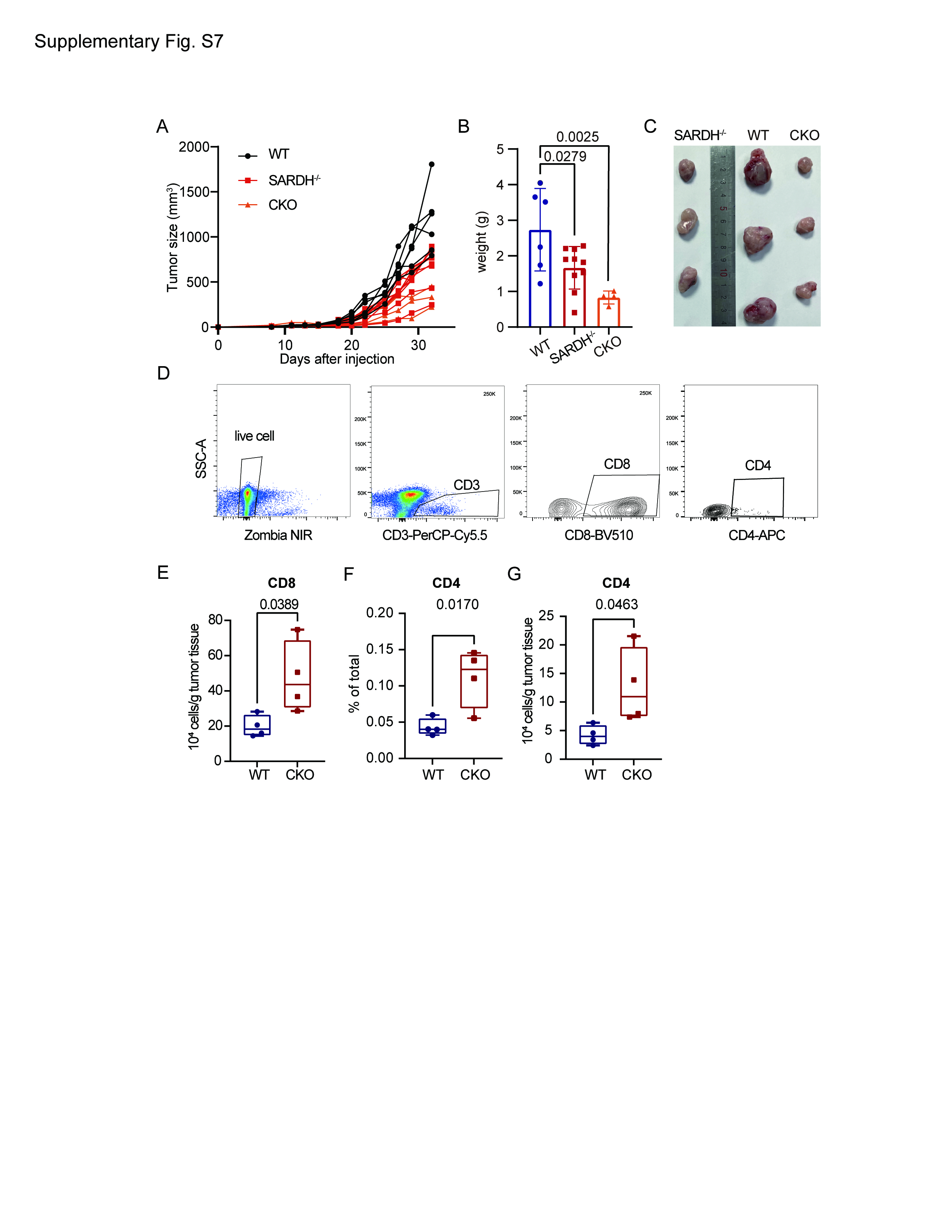

Supplement: Supplementary file 7 — Fig. S7 SARDH impairs the tumor control ability of T cells in vivo, related to Fig. 4 [file 41423_2025_1331_MOESM7_ESM.jpg]

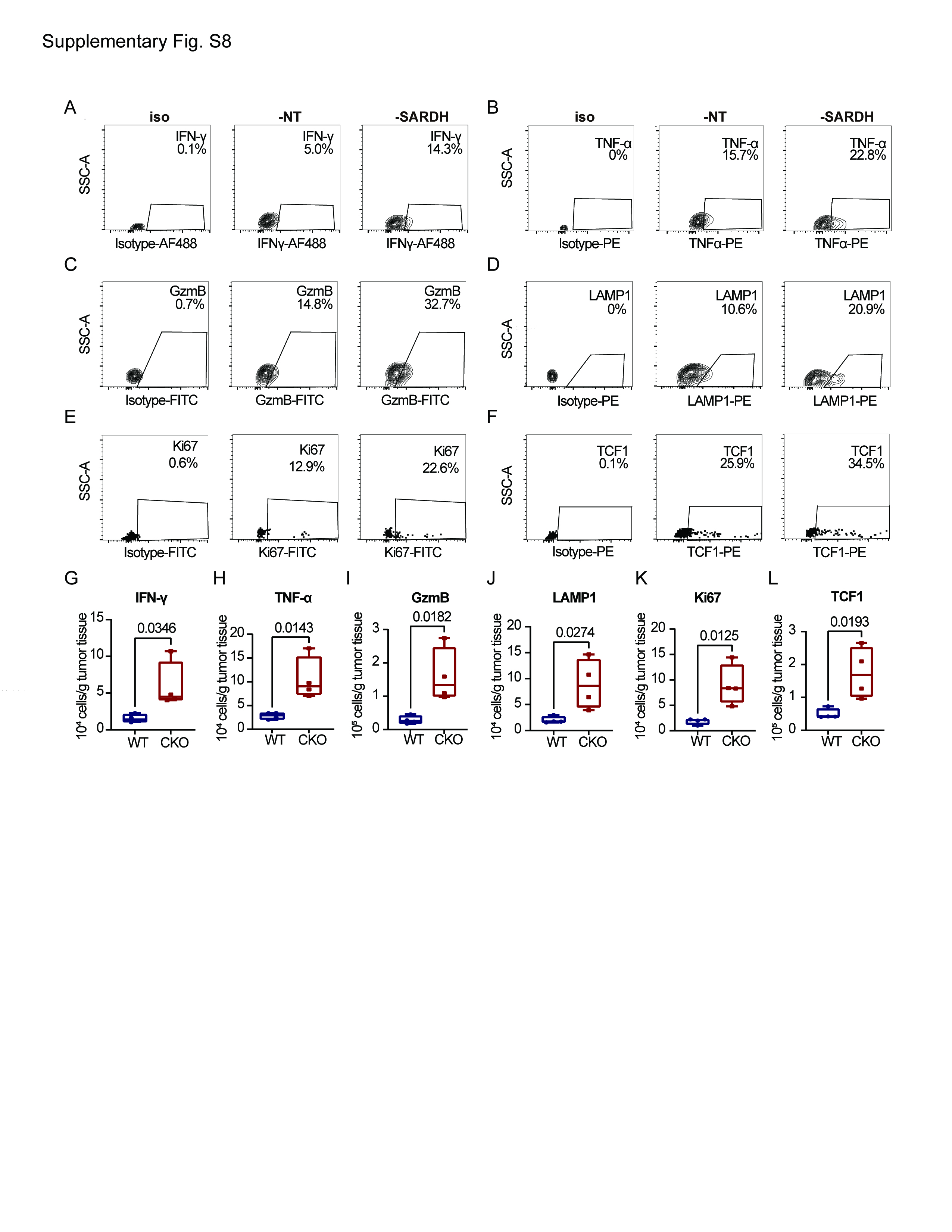

Supplement: Supplementary file 8 — Fig. S8. SARDH impairs the CD8+ T cell properties in vivo, related to Fig. 4 [file 41423_2025_1331_MOESM8_ESM.jpg]

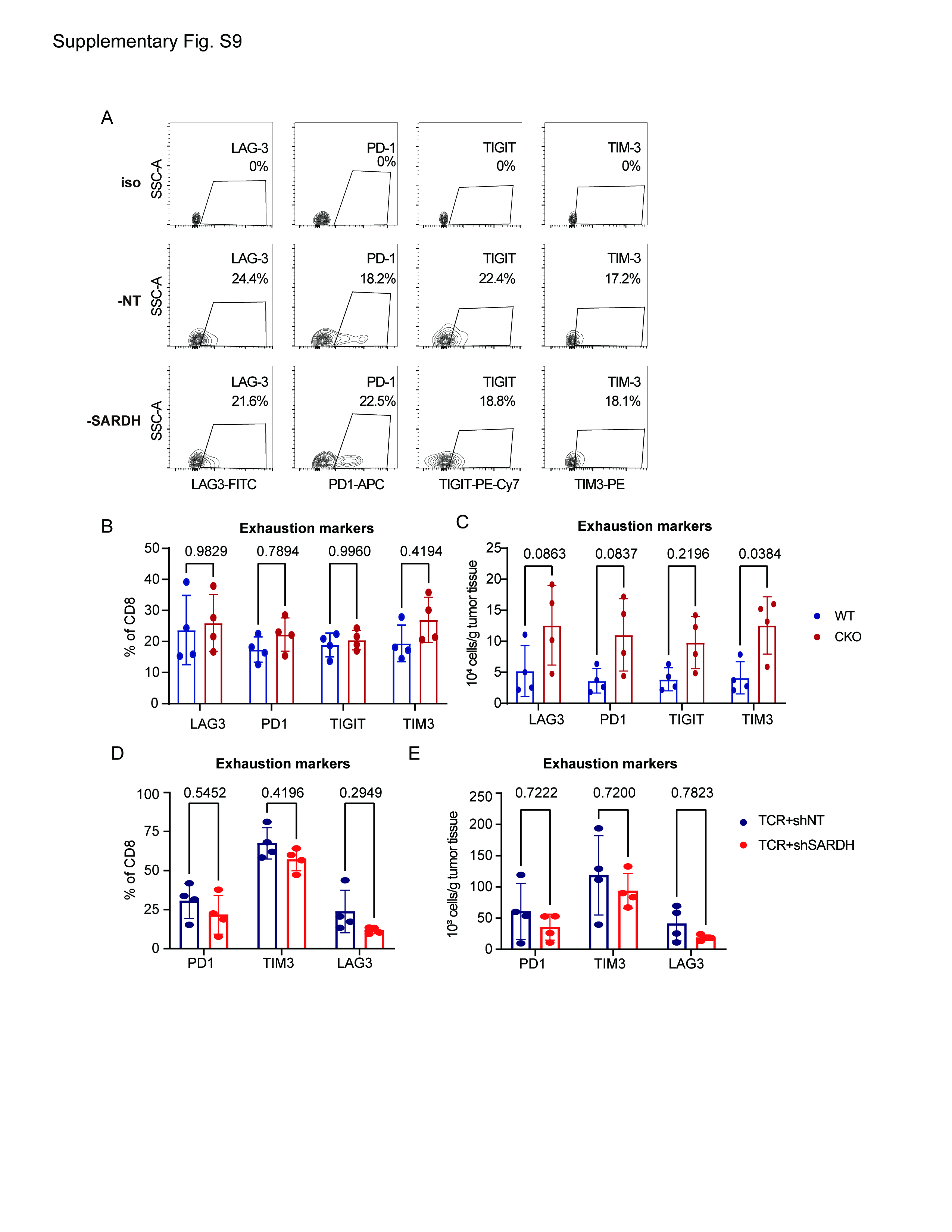

Supplement: Supplementary file 9 — Fig. S9 SARDH influences CD8+ T cell differentiation in vivo, related to Fig. 4 [file 41423_2025_1331_MOESM9_ESM.jpg]

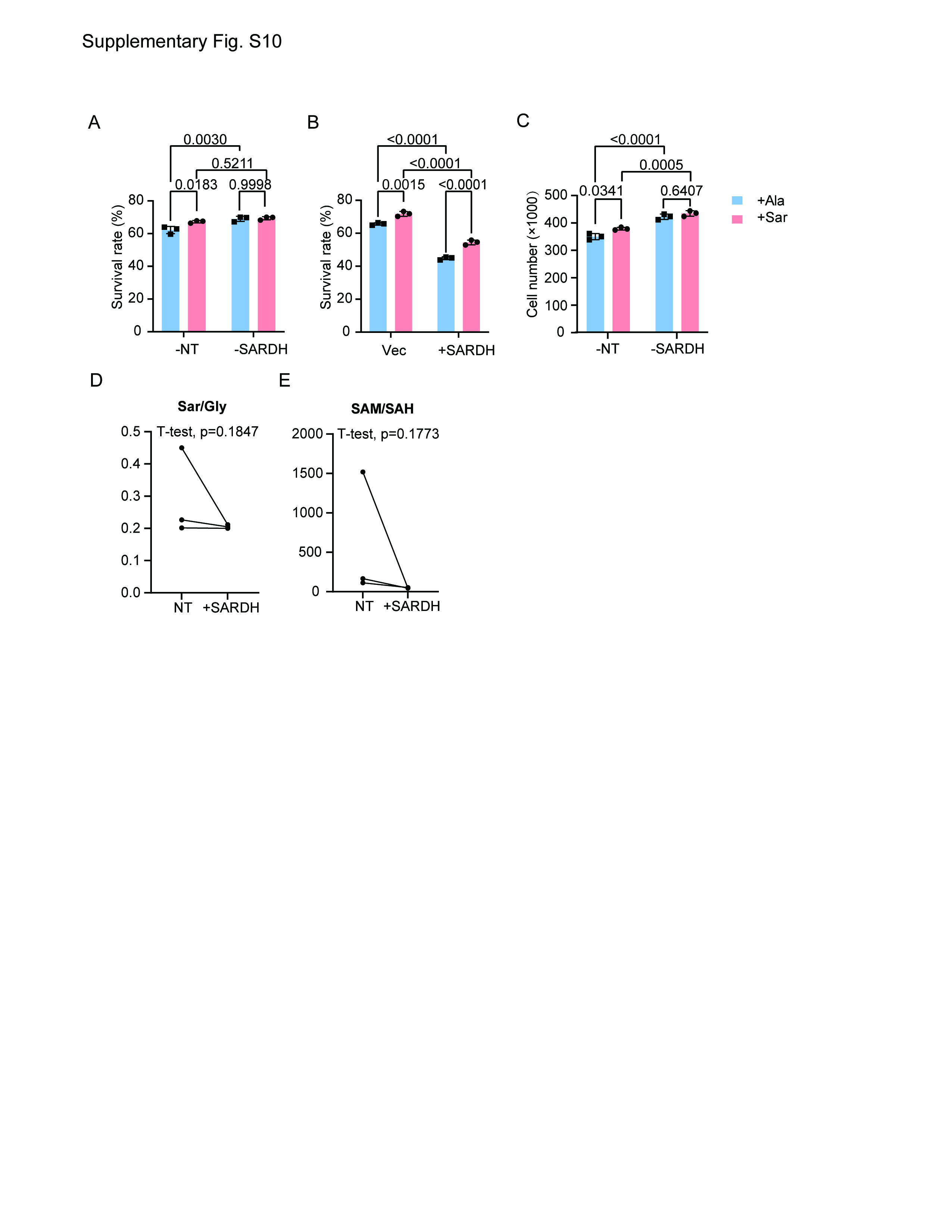

Supplement: Supplementary file 10 — Fig. S10 SARDH inhibits pathway-related cell properties by modulating the related metabolites, related to Fig. 5 [file 41423_2025_1331_MOESM10_ESM.jpg]

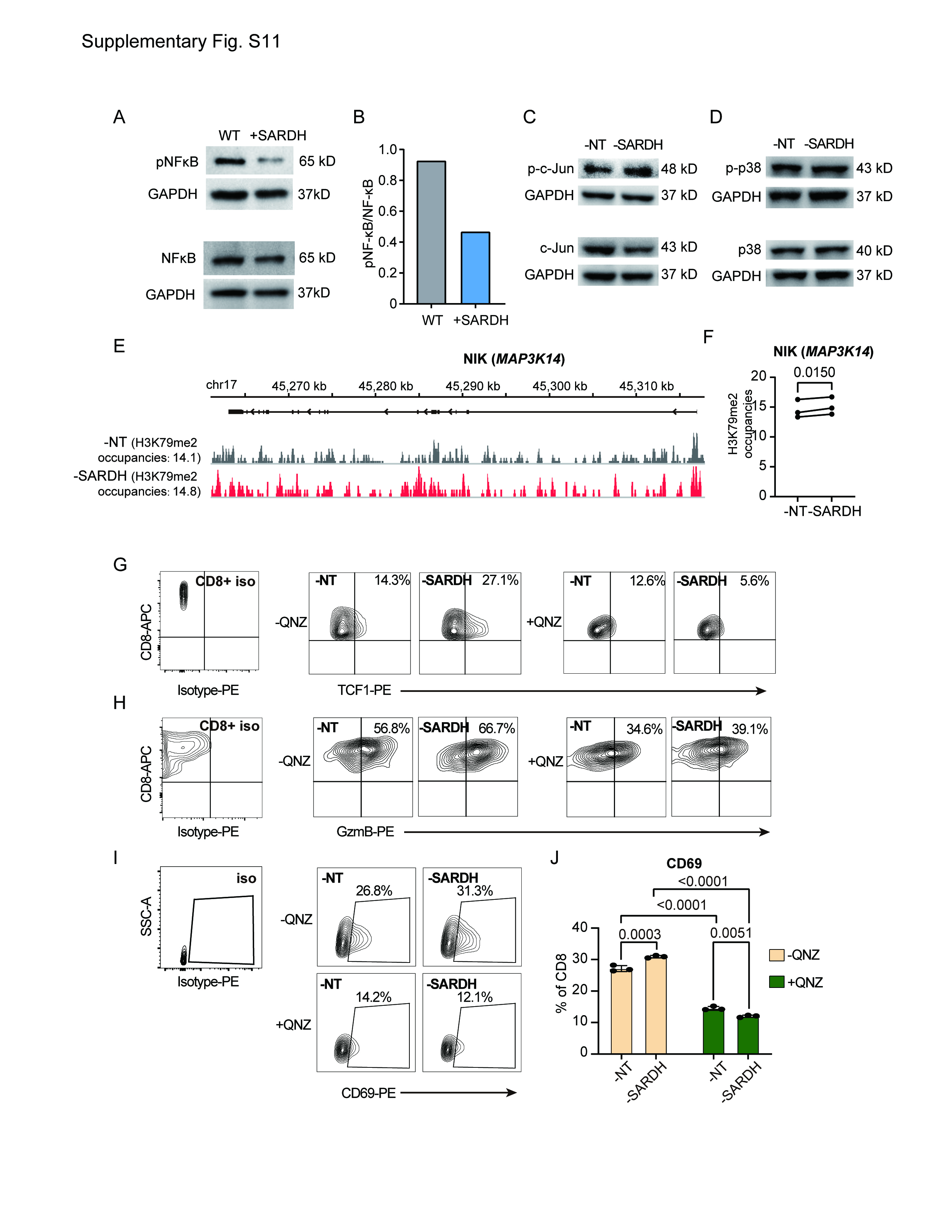

Supplement: Supplementary file 11 — Fig. S11 SARDH modulates T-cell function via methylation-dependent NF-κB inhibition, related to Fig. 6 [file 41423_2025_1331_MOESM11_ESM.jpg]

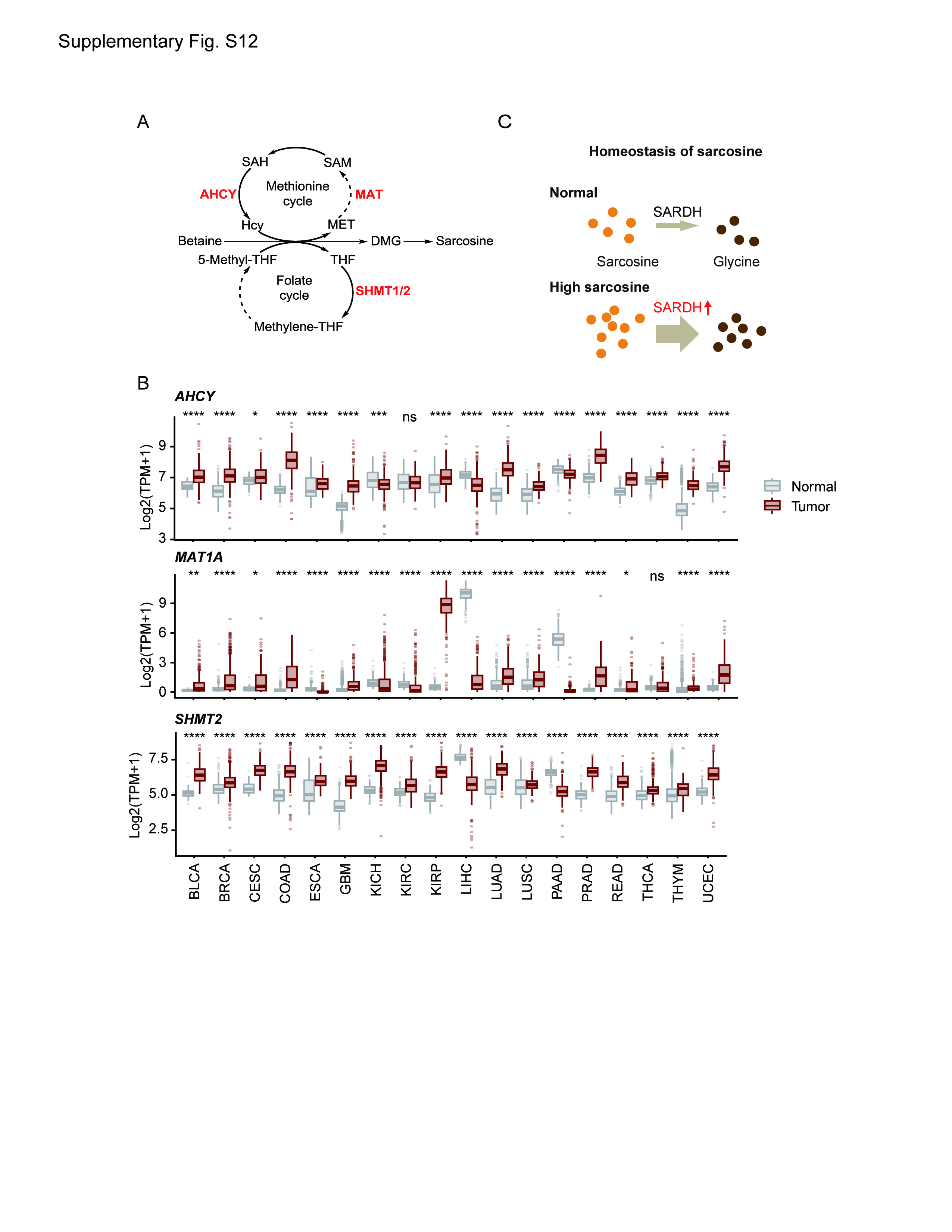

Supplement: Supplementary file 12 — Fig. S12 Dysregulation of 1-C metabolism in tumor, related to Fig. 7 [file 41423_2025_1331_MOESM12_ESM.jpg]
