# Supplementary material for: SARDH in the 1-C metabolism sculpts the T-cell fate and serves as a potential cancer therapeutic target
Source: Cell Mol Immunol. 2025 Aug 20;22(11):1363–78. doi: 10.1038/s41423-025-01331-5 (PMC12575850; doi:10.1038/s41423-025-01331-5)
Supplement: Supplementary file 13 — Supplementary figure Legend [file 41423_2025_1331_MOESM13_ESM.docx]

**Fig. S1 1-C metabolism is reprogrammed during tumor infiltration, and *SARDH* is specifically enriched in exhausted T cells (related to Fig. 1)**

**A** Histogram illustrating the distribution of the number of significantly dysregulated pathways across different numbers of cancer types. **B** Venn diagram showing the number of mitochondrial genes in the MitoCarta, MitoProteome, and Amigo gene sets. **C** Scatterplot illustrating the average log_2_(fold changes) and enrichment scores of mitochondrial genes that are significantly upregulated across 18 cancer types. **D** Scatterplot showing the average correlation coefficients with exhaustion markers and exhaustion scores of mitochondrial genes significantly upregulated in 18 cancer types. **E** Violin plot showing the expression of *SARDH* in each CD8^+^ subtype. **F** Violin plot showing the expression of *SARDH* in each CD4^+^ subtype. **G** Scatterplot illustrating the ratio of gene expression levels in T cells to the median expression value across all normal cell types, along with the ranking of gene expression levels within T cells compared with all normal cell types. Only the top 8 genes with the highest enrichment and exhaustion scores, as indicated by the genes enclosed in the dashed box of Fig. 1D, were plotted. **H** Bar plot illustrating the distribution of cell types above or below the expression threshold, defined as 10% of the maximum TPM (transcripts per million) value among all the cell types for each gene.

**Fig. S2 Knocking down SARDH with RNAi, related to Fig. 2**

**A** FACS plots depicting the transfection efficiency of siNT and siSARDH, with FAM fluorescence used as a marker. **B** FACS plots depicting the transfection efficiency of shNT and shSARDH. EGFP was coexpressed with shRNA, and its fluorescence was used to indicate the transfection efficiency. **C** Western blot analysis of SARDH expression in nontargeting control and SARDH-knockdown human T cells, with both GAPDH and β-actin used as loading controls. **D** Bar plot showing the geometric mean fluorescence intensity of nontargeting control and SARDH-knockdown human T cells labeled with tetramethylrhodamine ethyl ester (TMRE). n = 4. The data are presented as the means with SDs. The significances presented were calculated via an unpaired t test.

**Fig. S3 SARDH restricts T-cell properties, related to Fig. 2**

**A** FACS plots depicting the gating strategy for identifying human CD8^+^ and CD4^+^ T cells. **B** Flow cytometry plots depicting the identification of CD69-expressing cells in nontargeting control and SARDH knockdown human CD8^+^ and CD4^+^ T cells. **C** Bar plot showing the top 20 GO terms with the lowest adjusted p values enriched in the SARDH-knockdown samples compared with the normal samples. The numbers in the solid circles indicate gene counts matched to the corresponding terms. **D-E** Flow cytometry plots depicting the identification of IFN-γ (**D**)- and TNF-α (**E**)-expressing cells in nontargeting control and SARDH-knockdown human CD8^+^ and CD4^+^ T cells. **F-G** Bar plots depicting the proportions of IFN-γ- (**F**) and TNF-α-expressing (**G**) cells among nontargeting control and SARDH-knockdown human CD4^+^ T cells. n = 3. The plotted data are presented as the means with SDs and are representative of three independent experiments. The significances presented were calculated via an unpaired t test. **H** Flow cytometry plots depicting the identification of granzyme B-expressing nontargeting control and SARDH-knockdown human CD8^+^ T cells.

**Fig. S4 SARDH restricts T-cell cytotoxicity, related to Fig. 2**

**A** FACS plots depicting the gating strategy for quantifying the killing efficiency of T cells. The relative number of target cells (A375 or T2) was quantified on the basis of the number of counting beads. **B** FACS plots depicting the overexpression efficiency of SARDH, with RFP fluorescence coexpressed to indicate the transfection efficiency. **C** Western blot analysis of SARDH expression in WT control and SARDH-overexpressing human T cells. **D** Bar plot showing the densitometric analysis results, which were used to quantify the level of SARDH normalized to that of GAPDH. **E** Relative number of A375 cells after treatment with nontargeting control, SARDH-knockdown, or SARDH-overexpressing T cells. n = 3.

The plotted data are presented as the means with SDs and are representative of at least three independent experiments. The significances presented were calculated via an unpaired t test.

**Fig. S5 SARDH influences T-cell differentiation and proliferation, related to Fig. 2**

**A** Flow cytometry plots depicting the expression of exhaustion markers, including PD-1, Tim-3 and Lag-3, based on the isotype control in nontargeting control and SARDH-knockdown human CD8^+^ T cells. **B** Scatterplot showing the expression of exhaustion-related genes in the SARDH-knockdown samples compared with the nontargeting controls. n = 5. **C** FACS plots illustrating the percentages of Tcm (defined as the CCR7^+^CD45RA^-^ subtype) cells among nontargeting control and SARDH-knockdown human CD4^+^ T cells. **D** Relative quantification of Tcm percentages in nontargeting control and SARDH-knockdown human CD4^+^ T cells. n = 3. **E** Flow cytometry plot showing the staining of human CD8^+^ T cells with the isotype control for the TCF-1 antibody. **F** FACS plots illustrating TCF-1 expression in human CD4^+^ T cells in the nontargeting control and SARDH knockdown groups. **G** Quantification of the percentages of TCF-1-expressing nontargeting control and SARDH-knockdown human CD4^+^ T cells. n = 3. **H** FACS histogram showing fluorescence intensity distributions in CFSE-stained nontargeting control and SARDH-knockdown human CD4^+^ T cells. **I** Quantification of the proliferation abilities of nontargeting control and SARDH-knockdown human CD4^+^ T cells. n = 3.

All the bar plot data are presented as the means with SDs and are representative of at least three independent experiments. All significances presented were calculated via an unpaired t test unless otherwise indicated in the figure.

**Fig. S6 SARDH inhibits the migration and infiltration of T cells (related to Fig. 3)**

**A** Bar plot showing the migration status of human T cells after 9 hours in both the nontargeting control and SARDH knockdown groups. n = 3. **B** Bar plot showing GO enriched adhesion-related terms in the SARDH-knockdown samples compared with the nontargeting controls. The numbers in the solid circles represent gene counts matched to corresponding biological pathways. **C** Images of a representative coculture system after 48 hours of coculture with tdTomato^+^ LLC-OVA spheroids (red) and stained SARDH^+/+^ or SARDH^-/-^ OT-1^+^ CD8^+^ T cells (green). **D** Scatter dot plot illustrating the infiltration of SARDH^+/+^ or SARDH^-/-^ OT-1^+^ CD8^+^ T cells into LLC-OVA spheroids. n = 5. **E** Bright-field and multispectral fluorescence images of a representative coculture system after 48 hours of coculture with MART-1^+^ EGFP^+^ A375 spheroids (green) and nontargeting control or SARDH-knockdown human TCR-T cells (red). **F** Scatter dot plot illustrating the infiltration of nontargeting control and *SARDH*-knockdown human T cells into MART-1^+^ EGFP^+^ A375 spheroids. n = 8. **G** Images of the primary tumor organoid (stained with CFSE) induced from a patient with colorectal cancer (CRC). **H** Images of the primary tumor organoid and T-cell coculture system (48-hour coculture). **I** Three-dimensional reconstruction images after 48 hours of coculture of stained tumor cells (green) and T cells (red) from a colorectal cancer (CRC) patient via a two-photon microscope (upper panel), alongside the corresponding visualization of the spheroid with infiltrated T cells without showing the cells beyond the spheroid boundary (lower panel). **J** Scatter dot plot illustrating the infiltration of nontargeting control and SARDH-knockdown CRC patient T cells into primary spheroids. n = 4.

All the bar plot data are presented as the means with SDs, and all the scatter dot plot data are presented as the medians with interquartile ranges. All the data shown are representative of at least three independent experiments. All significances presented were calculated via an unpaired t test.

**Fig. S7 SARDH impairs the tumor control ability of T cells *in vivo* (related to Fig. 4)**

**A** Tumor growth curves of WT, SARDH^-/-^, and CKO OT-1^+^ mouse individuals. **B** Bar plot showing the tumor weight distributions of WT, SARDH^-/-^ and CKO OT-1^+^ mice. WT: n = 6; SARDH^-/-^: n = 10; CKO: n = 4. **C** Representative image of tumors from WT, SARDH^-/-^ and CKO OT-1^+^ mice. **D** FACS plots depicting the gating strategy for mouse CD8^+^ and CD4^+^ T cells. **E** Box-and-whisker plot showing the number of CD8^+^ T cells per gram of tumor tissue in WT and SARDH^-/-^ OT-1^+^ mice. **F** Box-and-whisker plot revealing the quantification of CD4^+^ T-cell percentages in tumors from WT and CKO OT-1^+^ mice. n = 4. **G** Box-and-whisker plot showing the number of CD4^+^ T cells per gram of tumor tissue in WT and SARDH^-/-^ OT-1^+^ mice.

All the bar plot data are presented as the means with SDs, and all the scatter dot plot data are presented as the medians with interquartile ranges. All the data shown are representative of at least three independent experiments. All significances presented were calculated via an unpaired t test.

**Fig. S8. SARDH impairs CD8^+^ T-cell properties *in vivo*, related to Fig. 4**

**A‒F** Flow cytometry plots of the expression of markers, including IFN-γ (**A**), TNF-α (**B**), granzyme B (**C**), Lamp-1 (**D**), Ki67 (**E**) and TCF-1 (**F**), in tumor-infiltrating CD8^+^ T cells isolated from WT and CKO mice. **G-H** Box-and-whisker plots quantifying the number of IFN-γ^+^ (**G**), TNF-α^+^ (**H**), granzyme B^+^ (**I**), Lamp-1^+^ (**J**), Ki67^+^ (**K**) and TCF-1^+^ (**L**) CD8^+^ T cells per gram of tumor tissue in WT and SARDH^-/-^ OT-1^+^ mice. n = 4.

The data are presented as the means with SDs and are representative of at least three independent experiments. All significances presented were calculated via an unpaired t test.

**Fig. S9 SARDH influences CD8^+^ T-cell differentiation *in vivo* (related to Fig. 4)**

**A** Flow cytometry plots depicting the identification of cell exhaustion markers, including LAG-3, PD-1, TIGHT, and TIM-3, in tumor-infiltrating CD8^+^ T cells isolated from WT and CKO mice. **B** Percentages of CD8^+^ T cells expressing the exhaustion markers LAG-3, PD-1, TIGHT, and TIM-3 in WT and CKO CD8^+^ T cells. n = 4. **C** Bar plots quantifying the number of CD8^+^ T cells expressing LAG-3, PD-1, TIGHT, and TIM-3 per gram in WT and SARDH^-/-^ OT-1^+^ mice. n = 4. **D** Percentages of CD8^+^ T cells expressing the exhaustion markers PD-1, TIM-3, and LAG-3 in the TCR-T mouse model. n = 4. **E** Bar plots quantifying the number of CD8^+^ T cells expressing PD-1, TIM-3, or LAG-3 per gram of tumor tissue in the TCR-T-cell-based mouse model. n = 4.

The data are presented as the means with SDs and are representative of at least three independent experiments. All significances presented were calculated via an unpaired t test.

**Fig. S10 SARDH inhibits pathway-related cell properties** **by modulating related metabolites (related to Fig. 5)**

**A** Bar plot showing the survival rates of nontargeting control and SARDH-knockdown human T cells treated with sarcosine (+Sar) or the control amino acid alanine (+Ala). n = 3. **B** Bar plot showing the survival rates of control (Vec) and SARDH-overexpressing (+SARDH) human T cells treated with sarcosine or alanine. n = 3. **C** Numbers of nontargeting control and SARDH-knockdown human T cells treated with sarcosine or alanine. n = 3. **D-E** Scatterplots illustrating the ratios of sarcosine to glycine (**D**) and SAM to SAH (**E**) in nontargeting control and SARDH-knockdown human CD8^+^ T cells. The significance presented was calculated via a paired one-sided t test. n = 3.

All the bar plot data are presented as the means with SDs and are representative of at least three independent experiments. All significances presented in the bar plots were calculated via an unpaired t test.

**Fig. S11 SARDH modulates T-cell function via methylation-dependent NF-κB inhibition, related to Fig. 6**

**A** Western blot analysis of p65 and phosphorylated p65 levels in wild-type control (WT) and SARDH-overexpressing (+SARDH) human T cells (12 days after cell activation). **B** Bar plot showing the densitometric analysis, which was used to quantify NF-κB activation (phosphorylation levels of p65) in WT and +SARDH human T cells. **C** Western blot analysis of c-Jun and phosphorylated c-Jun (Ser73) levels in nontargeting control and SARDH-knockdown human T cells (12 days after cell activation). **D** Western blot analysis of p38 and phosphorylated p38 (Thr180/Tyr182) levels in nontargeting control and SARDH-knockdown human T cells (12 days after cell activation). **E** Scatterplot showing the quantification of H3K79me2 occupancy within the *MAP3K14* gene. The significance presented was calculated via a paired one-sided t test. n=3. **F** Genome browser view of the *MAP3K14* gene region via IGV. **G-I** Flow cytometry plots illustrating the percentages of TCF-1 (**G**)-, granzyme B (**H**)- and CD69 (**I**)-expressing cells among nontargeting control and SARDH-knockdown human CD8^+^ T cells with or without the addition of QNZ. **J** Bar plot depicting the percentages of human CD8^+^ T cells expressing CD69 in the nontargeting control and SARDH knockdown groups with or without the addition of QNZ. n = 3.

The data are presented as the means with SDs and are representative of at least three independent experiments. All significances presented were calculated via an unpaired t test unless otherwise indicated.

**Fig. S12 Dysregulation of 1-C metabolism in tumors, related to Fig. 7**

**A** Schematic representation of the upstream metabolic pathway related to sarcosine generation, illustrating key enzymatic steps and metabolites involved in the biosynthesis process. **B** Boxplot showing the comparison of *AHCY*, *MAT1A*, and *SHMT2* expression levels in tumor and normal tissues. All the statistically significant differences were calculated via Wilcoxon’s test and are shown in the Supplementary Table. n = 30. **C** Schematic depicting the mechanism by which sarcosine induces SARDH upregulation to maintain sarcosine homeostasis
